# ZO-1 and IL-1RAP Phosphorylation: Potential Role in Mediated Brain-Gut Axis Dysregulation in Irritable Bowel Syndrome-like Stressed Mice

**DOI:** 10.7150/ijms.95848

**Published:** 2024-07-02

**Authors:** Yu-Qin He, Jian-Ru Zhu, Wen-Jing Sun, Yuan-Yuan Luo, Xiao-Feng Wu, Min Yang, Dong-Feng Chen

**Affiliations:** 1 Gastroenterology, Chongqing Key Laboratory of Digestive Malignancies, Daping Hospital, Army Medical University (Third Military Medical University), Chongqing, 400042, China.; 2 Department of Gastroenterology and Hepatology, The Thirteenth People's Hospital of Chongqing, 400030, China.; 3 Key Laboratory of Biorheological Science and Technology, Ministry of Education, College of Bioengineering, Chongqing University, Chongqing, 400030, China.; 4 Department of Stem Cell and Regenerative Medicine, Gastroenterology, Daping Hospital, Army Medical University, Chongqing, 400042, China.

**Keywords:** IL1 receptor accessory protein (IL-1RAP), IBS-like symptoms, Tight junction protein ZO-1, phosphoproteomics

## Abstract

**Background and Objectives:** Irritable Bowel Syndrome (IBS) is a common gastrointestinal disorder often exacerbated by stress, influencing the brain-gut axis (BGA). BGA dysregulation, disrupted intestinal barrier function, altered visceral sensitivity and immune imbalance defects underlying IBS pathogenesis have been emphasized in recent investigations. Phosphoproteomics reveals unique phosphorylation details resulting from environmental stress. Here, we employ phosphoproteomics to explore the molecular mechanisms underlying IBS-like symptoms, mainly focusing on the role of ZO-1 and IL-1RAP phosphorylation.

**Materials and Methods:** Morris water maze (MWM) was used to evaluate memory function for single prolonged stress (SPS). To assess visceral hypersensitivity of IBS-like symptoms, use the Abdominal withdrawal reflex (AWR). Colonic bead expulsion and defecation were used to determine fecal characteristics of the IBS-like symptoms. Then, we applied a phosphoproteomic approach to BGA research to discover the molecular mechanisms underlying the process of visceral hypersensitivity in IBS-like mice following SPS. ZO-1, p-S179-ZO1, IL-1RAP, p-S566-IL-1RAP and GFAP levels in BGA were measured by western blotting, immunofluorescence staining, and enzyme-linked immunosorbent assay to validate phosphorylation quantification. Fluorescein isothiocyanate-dextran 4000 and electron-microscopy were performed to observe the structure and function of the intestinal epithelial barrier.

**Results:** The SPS group showed changes in learning and memory ability. SPS exposure affects visceral hypersensitivity, increased fecal water content, and significant diarrheal symptoms. Phosphoproteomic analysis displayed that p-S179-ZO1 and p-S566-IL-1RAP were significantly differentially expressed following SPS. In addition, p-S179-ZO1 was reduced in mice's DRG, colon, small intestine, spinal and hippocampus and intestinal epithelial permeability was increased. GFAP, IL-1β and p-S566-IL-1RAP were also increased at the same levels in the BGA. And IL-1β showed no significant difference was observed in serum. Our findings reveal substantial alterations in ZO-1 and IL-1RAP phosphorylation, correlating with increased epithelial permeability and immune imbalance.

**Conclusions:** Overall, decreased p-S179-ZO1 and increased p-S566-IL-1RAP on the BGA result in changes to tight junction structure, compromising the structure and function of the intestinal epithelial barrier and exacerbating immune imbalance in IBS-like stressed mice.

## Introduction

Irritable bowel syndrome (IBS) is a gastrointestinal ailment often linked to chronic diarrhea, constipation, abdominal pain and intermittent gastrointestinal dysfunction, with no known biomarker to date. The Rome Foundation working group reviewed the morbidity rates of IBS, ranging from 1.1% to 35.5% worldwide, and the incidence varies in Asia 1, 2. The exact pathophysiology of IBS remains unknown. Visceral hypersensitivity is a hallmark symptom in patients who show the symptoms of chronically persistent abdominal pain, and is often associated with abnormal bowel movements 3. Still, there is growing evidence of the involvement in both central and peripheral mechanisms, giving rise to the proposals for the brain-gut axis (BGA) 4-7. Stress, a known trigger for the development of visceral hypersensitivity in IBS, can have a significant impact on BGA 8. IBS patients who suffered from severe gastrointestinal symptoms were also reported to have various degrees of stress and related psychological reactions, such as depression 6, 9. Significant effects of stress on different biological functions of the gastrointestinal include: 1) visceral sensitivity; 2) permeability; 3) modifications in gastrointestinal secretion; 4) gastrointestinal motility; 5) intestinal microbiota 10-13.

In recent years, the pathogenesis of visceral hypersensitivity and diarrhea in IBS implicate the dysregulation of permeability in the intestinal epithelial barrier (IEB) 14, 15. Literature increasingly shows that the regulation of the IEB is highly influenced by components of both its 'outer' (microbiota, metabolites, and nutrients) and 'inner' microenvironments (stromal cells and immune cells). So the IEB interacts with the enteric nervous system and modulates visceral hypersensitivity functions. By analogy with 'Neuronal-Glial-Endothelial Unit' in the brain, a novel concept of a digestive 'Neuronal-Glial-Epithelial Unit' was put forward 16. The hypothesis is that the interplay of epithelial barrier dysfunction and consequent increased intestinal barrier permeability can allow microbial antigens' adherence, transport, and entrance into the mucosal barrier, triggering activation of the immune response of visceral hypersensitivity and diarrhea 17, 18.

Stress exacerbates IBS symptoms by directly signaling the colonic nociceptive dorsal root ganglion (DRG) 19-21. DRG is an essential mediator in visceral hypersensitivity 22 and can carry sensory signals from the peripheral to the central nervous system. However, whether and how DRG links epithelial barrier dysfunction and BGA in visceral hypersensitivity of IBS-like symptoms following stress remains unclear. Research on the underlying molecular mechanisms of visceral hypersensitivity and IBS-like symptoms following stress is scarce. Therefore, an integrative method is essential to unravel the molecular changes elicited by IBS-like symptoms following stress.

In recent years, phosphoproteomic analysis using mass spectrometry (MS) approaches facilitated the detection of widespread phosphorylation changes and revealed unique phosphorylation details resulting from environmental stress and signals 23. Besides, the isobaric tags for relative and absolute quantitation (iTRAQ) proteomics have proven effective in accurately characterizing and quantifying variations in total protein expression 24. This study utilized a quantitative phosphoproteomic approach using iTRAQ to investigate the phosphoproteomic profiles in IBS-like stressed mice. Our findings may provide a novel understanding of the molecular mechanisms underlying IBS-like symptoms following stress.

## Material and Methods

### Animals and development of SPS models

Male C57BL/6 mice (aged 5-6 weeks, weighing 18-28g) were purchased from Daping Hospital at the Army Medical University. The mice were kept in controlled conditions (21-25 °C, with a 12/12-hour dark/light cycle) and had unrestricted access to food and water. Before the experiments, the mice were fasted for 20-22 hours, although they were allowed unlimited access to clean drinking water. All animal experiments were conducted using institutional animal welfare policies. The Academic Animal Care and Advisory Group approved all animal experimental protocols at the Army Medical University in Chongqing, China (Approval No. AMUWEC20199001). The mice received excellent care and were handled in line with the International Association for the Study guidelines for Pain. To ensure they acclimated to their new environment, the mice were housed for a minimum of one week before any experiments were conducted. During the behavioral testing, the researcher remained blinded to the animal groupings.

The room where the mice were kept was cleaned every 3 days with peroxyacetic acid (0.1%). The mice cages and drinking water bottles were disinfected monthly and autoclaved every week. Soft food was replaced daily, and their drinking water was regularly autoclaved. Feces were removed from the cages daily. One week before surgery, each mouse was housed individually in a cage with unlimited access to food and water. We monitored their daily food and water intake. Before the experiment, they were anesthetized with an intraperitoneal injection of pentobarbital sodium (2%, 40 mg/kg) following isoflurane induction. Upon conclusion of our experiments, all participating mice were euthanized to ensure data accuracy and completeness. This was done to prevent any unnecessary suffering or stress after the experiments. For mice that needed to be sampled, we euthanized them via intraperitoneal injection of an overdose of pentobarbital sodium (100mg/kg). Mice used for behavioral testing were euthanized via cervical dislocation.

Previous studies have induced visceral hypersensitivity in IBS through different stress methods, such as water-avoidance stress 25, restraint stress 26, 27, and early-life stress 28. Some evidence suggests that IBS patients have a higher likelihood of being burdened with post-traumatic stress disorder (PTSD), and PTSD is accompanied by an increased probability of developing IBS 8, 29, 30. In current years, the Single prolonged stress (SPS) model of PTSD attempts to imitate serial exposure to numerous stressors from a single traumatic experience through psychological (restraint), physiological (forced swim), and pharmacological (anesthesia). This model has been confirmed to independently activate behavioral and neurobiological manifestations and the HPA axis in C57BL/6 mice and rats following a 7-day 'no-touch' sensitization period 31-35. SPS was performed following the method described in 36. 1^st^, the mouse was restricted (immobilization 2 hours) in a Falcon 50 mL tube. 2^nd^, the mouse was compelled to swim exhaustively in a cylinder made of plastic (height: 21 cm; diameter: 13 cm) with water (21 to 24 °C). 3rd, with a 15-minute break, the animals were given anesthetic isoflurane (5%) via inhalation till unconsciousness (no more than 5 min). To evaluate stressed mice, we used behavioral tests, such as Morris Water Maze (MWM), stool collection, and visceral sensitivity to the abdominal withdrawal reflex (AWR).

### Morris water maze examination

As previously described, the Morris Water Maze (MWM) was used to assess spatial learning and memory function in control and SPS mice 37. The MWM facilities included a round pool (160 cm, diameter; 55 cm, height) categorized into 4 equal-sized segments, with opaque water at approximately 23 cm of water dyed with black ink at 22±2 °C. In one of the segments, a cylindrical black platform (10 cm diameter and 21 cm height) was installed (the target quadrant). During learning experiments in the southwest quadrant, a black target platform (diameter 8 cm) was submerged (about 1 cm) under the water. A computer-controlled video camera was linked to a digital video camera device and placed immediately over the water to capture all swimming workouts in various segments (Smart video-tracking system; Panlab, Barcelona, Spain). We released each mouse into one segment and allowed it to swim independently to swim independently for 60 seconds to discover the concealed platform. And then, we recorded the time spent finding the platform as the escape latency. Each mouse was given a 20-second break between experiments. If the animals did not discover the escape platform within 60 seconds, they were escorted and allowed to remain there for 20 seconds. The average value of escape latency time obtained from the 4 experiments on that day was calculated. After the experiment, the mice were returned to the cage for regular feeding. MWM assessment was performed from the 7^th^ to the 11^th^ day both in control and the SPS-treated groups to assess changes in spatial learning and memory. Each mouse was allowed to perform 4 times a day for 4 days in a row for learning performance.

### Stool collection and examination of the fecal water content

Over 24 hours, free-feeding mice were monitored, and the number of fecal pellets they produced was tallied for one specific hour, as referenced in 38. Fecal pellets were collected using paper towels. Following collection, any changes in the form of the fecal pellets were observed, recorded, and then the pellets were weighed. Animals were generally transferred from their housing to single clean cages without access to water, food, or bedding. Every hour, for 15 minutes, each animal's fecal pellets were collected in pre-weighed vials after being placed in the new environment. The fecal water content was determined by reassessing the vials after allowing the pellets to dry overnight in an oven at 65 °C. The following equation was used to compute the % water content: [(stool wet weight - stool dry weight)/ (stool wet weight)] × 100. All *in vivo* experiments were conducted between 9 a.m. and 12 a.m.

### Abdominal Withdrawal Reflex (AWR)

To measure visceral hypersensitivity following SPS by measuring the AWR to colorectal distension using a semiquantitative score 39-41. This method helps avoid potential confounding factors, such as stress caused by restraint, the use of anesthesia, and invasive surgical procedures for implanting electrodes. On the day before the test, mice were fasted but had free access to water. This helped to standardize the conditions for the experiment and minimize confounding factors related to digestion. These two sentences can be combined for fluency: “To minimize stress and ensure accurate catheter placement, mice were lightly sedated with ether during insertion. A double-lumen catheter (6-Fr, from Technology Development Yangzhou Huayue Co., Ltd., China) coated with paraffin oil was gradually inserted 2cm into the mouse's anus. The catheter was subsequently secured to the base of the mouse's tail using medical tape. Mice were allowed to fully recover from the sedation and placed in a transparent box with holes to restrict their movement while still allowing for observation. This setup minimized stress and provided a controlled environment for testing. After the mice had settled, one experimenter slowly administered normal saline (NS) into the balloon via a 1mL syringe, while another observed the mice for responses. The primary indicator of a pain reflex was the abdominal withdrawal reflex (AWR), manifested by the mouse lifting its abdomen or adopting a hunched posture with tail tightening. The volume of NS that elicited the pain reflex (tail tightness or abdominal wall tightening) was noted. Each expansion lasted 15 seconds and was followed by a 5-minute interval before the next repetition. This process was repeated three times, and the average volume required to trigger the pain reflex was calculated. The average volume of NS needed to induce the AWR was used to measure the visceral pain threshold. Higher volumes indicated a higher pain threshold, suggesting reduced visceral sensitivity, while lower volumes indicated increased sensitivity.

### Hematoxylin & Eosin (H&E) staining

The H&E staining was shown in the intestinal and colonic specimens of the two group mice to assess the degree of colitis. The intestinal and colonic tissues, which had been fixed in 4% formalin, were sectioned into 4 µm slices. Later, sample processing involved deparaffinization and rehydration of the paraffin-embedded tissue sections, a hematoxylin solution for 2 min stained the sections afterward, and 3 drops of acid ethanol (1%). Later, an eosin solution stained the slices. Finally, the slices were dehydrated with graded alcohol and xylene. The stained sections were observed and photographed under an Olympus microscope (version 2.2, Tokyo, Japan).

### Bead expulsion examination

Mice were exposed to the previously reported bead ejection assessment simultaneously after the fecal pellet output assessment. To facilitate the implantation of a bead, the animals were mildly sedated with 2% isoflurane. As mentioned, bead glass (3 mm) was introduced inside the colon (2 cm distal to the anus) with a pasteurized plastic gently greased using lubricant jelly. The time from bead implantation to ejection was precisely measured to the second 42.

### Phosphoproteome

#### Extraction and digesting of proteins

For specimen lysis and protein extraction, we used SDT lysis buffer at pH 7.6. We measure the protein content using the bicinchoninic acid Kit for Protein Assay (Bio-Rad, USA). Proteins were digested with trypsin using Matthias Mann's filter-aided sample preparation (FASP) method 43. Digest peptides from each sample were desalinated using C18 Cartridges (EmporeTM SPE Cartridges C18, with a usual volume of 3 ml, bed I.D. 7 mm, from Sigma). The peptides were then vacuum centrifuged and eluted with 0.1% formic acid (40 µl).

#### SDS-PAGE

We used a 12.5% SDS-PAGE gel (run for 90 minutes at a continuous current of 14 mA) to separate the 20 µg of proteins, which were heated for 5 min. Protein bands were visualized using Coomassie Blue R-250 staining.

#### Labeling

Tandem mass tags (TMTs)-labeled liquid chromatography-mass spectrometry (LC-MS/MS), a classical shotgun DDA-MS methodology, overcomes many problems associated with previously described isotope labeling techniques. It was used to analyze the phosphoproteome. The reliability of using a standardized collision energy for the same set of markers is ensured. Hence, the samples, consisting of 100 μg of peptide each, were labeled using TMT (Thermo Fisher Scientific, Waltham, USA).

#### Enrichment of phosphopeptides and LC-MS/MS

**TiO2 enrichment method:** The specimens were reconstructed in a pre-cooled immunoprecipitation buffer (1.4 mL). TiO2 beads were added to the specimen, which was agitated for 40 minutes before centrifuging and discarding the supernatant. The beads were then transferred to the pipette tips, washed using buffer A (3 times), and then using buffer B (3 times). To elute, the elution buffer contained phosphopeptides, which were then dried under vacuum and dissolved in FA (0.1%, 20 μL) for MS examination.

**LC-MS/MS examination:** For 120 minutes, an LC-MS/MS analysis was performed using a Q Exactive HF (Thermo Scientific) mass spectrometer coupled to an Easy nLC. In buffer A, the peptides were loaded onto a trap column in reverse phase connected to a reversed-phase C18 analytical column and then eluted using a gradient of buffer B. The MS analysis was conducted in positive ion mode. During the survey scan ranging from 300 to 1800 m/z, data for high-energy collision dissociation fragmentation was acquired using a data-dependent top 10 method that consistently selected the most abundant parent ions detected in the scan. Additionally, the instrument was configured to recognize and analyze peptides.

**Phosphorylated protein quantification and identification:** We utilize the MASCOT engine (Matrix Science, London, UK; version 2.2) to analyze MS/MS spectra integrated within Proteome Discoverer 2.4.

### Bioinformatic analysis

**Phosphorylated peptide cluster evaluation:** The hierarchical clustering process employed Java Treeview software (http://jtreeview.sourceforge.net) alongside Cluster 3.0. The Euclidean distance metric was used to measure similarity, and average linkage hierarchical clustering (clustering based on the centroids of observations) was applied. A heat map was employed as a visual aid to complement the dendrogram.

**Annotation for GO:** The sequences of the selected differentially expressed proteins (DEPs) were locally analyzed using the NCBI BLAST+ client software and InterProScan tool. Subsequently, GO terms were plotted, and the data were interpreted with the help of the software Blast2GO.

**Kyoto Encyclopedia of Genes and Genomes (KEGG) annotation:** To analyze biological processes, drug action mechanisms, and disease pathogenesis, we broadened our approach by considering a range of protein interaction modifications, including metabolic pathway adjustments. We compared the proteins under investigation with the online KEGG database (https://www.kegg.jp/) to obtain KEGG orthology identifications, which were then mapped to KEGG pathways.

**Analysis of Enrichment:** Enrichment analysis was performed utilizing the field effect transistor (FET) approach, using the entire protein quantification dataset as background. The Benjamini-Hochberg correction for multiple testing was also applied to adjust the calculated *p*-values. Furthermore, pathways or working groups with *p*<0.05 were considered statistically significant.

**Protein-protein interaction (PPI) studies:** Protein-protein interaction (PPI) data were obtained from the database of molecular interactions, IntAct (http://www.ebi.ac.uk/intact/), using their gene codes, or from the STRING software (http://string-db.org/). The PPI data obtained were in XGMML format and were imported into Cytoscape software (version 3.2.1, available at http://www.cytoscape.org/) to visualize and analyze the PPI networks. Additionally, within the PPI network, the connectivity or degree of each protein was determined to assess its relevance.

### Western blotting

Western blot analysis was performed as described in the literature 44. Every sample (100 mg) was homogenized in RIPA buffer (P0013B, Beyotime, 1 mL), including recently added PMSF (ST506, Beyotime, 1 mM) and protein phosphatase blockers (Beyotime, P0013B). Lysates were quickly homogenized and centrifuged (4 °C, 12,000 g) for 15 min. Supernatants were gathered and used to evaluate the protein content in tissue samples by a BCA kit (P0010S, Beyotime). The hippocampi, spinal, DRG, intestinal, and colon specimens (40 μg/lane) were uploaded on 8-10% SDS gels and PVDF membranes (Millipore Corp. Bedford, MA, USA). The blotting membranes were then blocked (2 hours) with skim milk (5%) containing Tween-20 in TBS (TBST, 0.1%). The membranes were incubated with diluted antibodies: Rabbit monoclonal to ZO1 tight junction protein [ZO1] (1:1000, Abcam ab276131), Goat Anti-Rabbit to IL-1RAP antibody (1:1000, Abcam ab256461), monoclonal antibodies from mice against GAPDH (1:1000, Abcam ab9485), Mouse monoclonal GFAP (1:500, Abcam, ab279289), phospho-IL-1RAP (S566), phospho-ZO1 (S179) (1:500, reagent customized from Sino Biological, Beijing, China) overnight (4 °C). Blots were washed with TBST (3 times) before incubating with a secondary antibody (2 hours). The blots were visualized using improved chemiluminescence. The similar blots were treated with GAPDH antibodies to demonstrate equivalent protein loading. ECL was used to evaluate GAPDH immunoreactivity. A ChemiDoc MP Imaging System (Bio-Rad, Hercules, CA, USA) was used to identify protein expression signals. The Gel Image Processing System was utilized to compute proteins' optical densities (ODs) (Tanon 2500R, Shanghai, PR China).

### Immunofluorescence

Following anesthesia, the animals were perfused using ice-cold heparinized PBS and 4% paraformaldehyde in PBS perfusion. In preparation for cryosectioning, brains were injected with the same fixative and then submerged in dissolved sucrose in 0.01 M PB (30%, pH 7.4, 4 °C). For immunofluorescence analysis, Liquid nitrogen was used to freeze the samples and sections were cut at 25 μm. The samples were blocked with 5% bovine serum albumin (2 hours). The perivascular localization of ZO1 and double immunofluorescence labeling were used to identify reactive astrocytes. The samples were incubated with diluted antibodies: Mouse monoclonal to GFAP (1:500, Abcam, ab279289) together with rabbit monoclonal to tight junction protein ZO1 (1:500, Abcam, ab221547), phospho-ZO1 (S179) and phospho-IL-1RAP (S566) (1:500, reagent customized from Sino Biological, Beijing, China) overnight (4 °C). Secondary antibodies Alexa 594-conjugated donkey anti-mouse IgG (1:200, Abcam 150108) and donkey anti-rabbit IgG (1:200, Abcam 150073) conjugated with Alexa 488 were supplementary to the slices for incubation (2 hours). Then 4',6-diamidino-2-phenylindole (DAPI) was applied for nuclear staining. The slices were photographed and analyzed using a fluorescent microscope and the software DP2-BSW (version 2.2, Olympus, Tokyo, Japan).

### Ultrastructural observation of colon

The colon tissues were perfused and fixed in a 2.5% glutaraldehyde solution for 24 hours for structural examination. The tissues should be kept away from light after being post-fixed with OsO4 (1%) in PB (0.1 M, pH 7.4) for 2 hours. Subsequently, after removing the OsO4, the tissues are washed in PB. Following dehydration, resin penetration, embedding, and polymerization, the resin blocks are sliced to 60nm thickness on an ultra-microtome. Uranium acetate (2%) in a saturated alcohol solution is used for staining in the dark (8 minutes), followed by rinsing in ethanol (70%, 3 times) and then in ultra-pure water (3 times). Lead citrate (2.6%) is used for CO2-free staining (8 minutes), followed by rinsing with ultra-pure water (3 times). The ultrastructure of colonic epithelial cells was observed using a transmission electron microscope (HITACHI, HT7800, 80 kV, China).

### Test for Fluorescein Isothiocyanate-Dextran 4000 (FITC-D4000)

The FITC-D4000 test is frequently used in IBS studies and has been extensively documented 45. The 4000-Da sample material may be easily identified in blood plasma using a photometric technique. FITC-D4000 (Sigma-Aldrich, St. Louis, MO) was administered via gastric lavage at 0.2 ml (50 mg/ml) into the mice. After 3.5 hours, the blood was collected from the mice, which was subsequently heparinized and centrifuged (12,000g, 10 min, 4 °C), and the plasma was light-shielded for photometric measurement of FITC-D4000. 100 µl aliquots of diluted animal samples, standards, and blanks (PBS and untreated animals' diluted plasma) were loaded onto black 96-well microplates (BKMAM, Wuhan, China). A spectrophotometer fluorescence (Microplate Reader, Multi-Detection, Synergy TM HT, Bio-Tek, Vermont, US) and software KC4 were used to determine the FITC-D4000 concentration (excitation: 485 nm, emission: 528 nm). IBS was estimated using a standard curve with PBS-diluted FITC-dextran (0, 125, 250, 500, 1000, 2000, 4000, 6000, and 8000 ng/mL amounts).

### Enzyme-Linked Immunosorbent Assay (ELISA)

Cytokines in the blood and tissue were measured by means of an ELISA, with kits for interleukin-1 beta (IL-1β) obtained from Servicebio (Wuhan, China), as recommended by the manufacturer.

### Statistical evaluation

The data is displayed as mean ± standard error of the mean (SEM). The relevant statistical methods are presented in the Figure legends using GraphPad Prism 8 software (GraphPad Software, Inc., San Diego, CA, USA). Differences were considered significant at *p*<0.05.

## Results

### SPS-induced memory and learning ability changes

**Figure 1:**
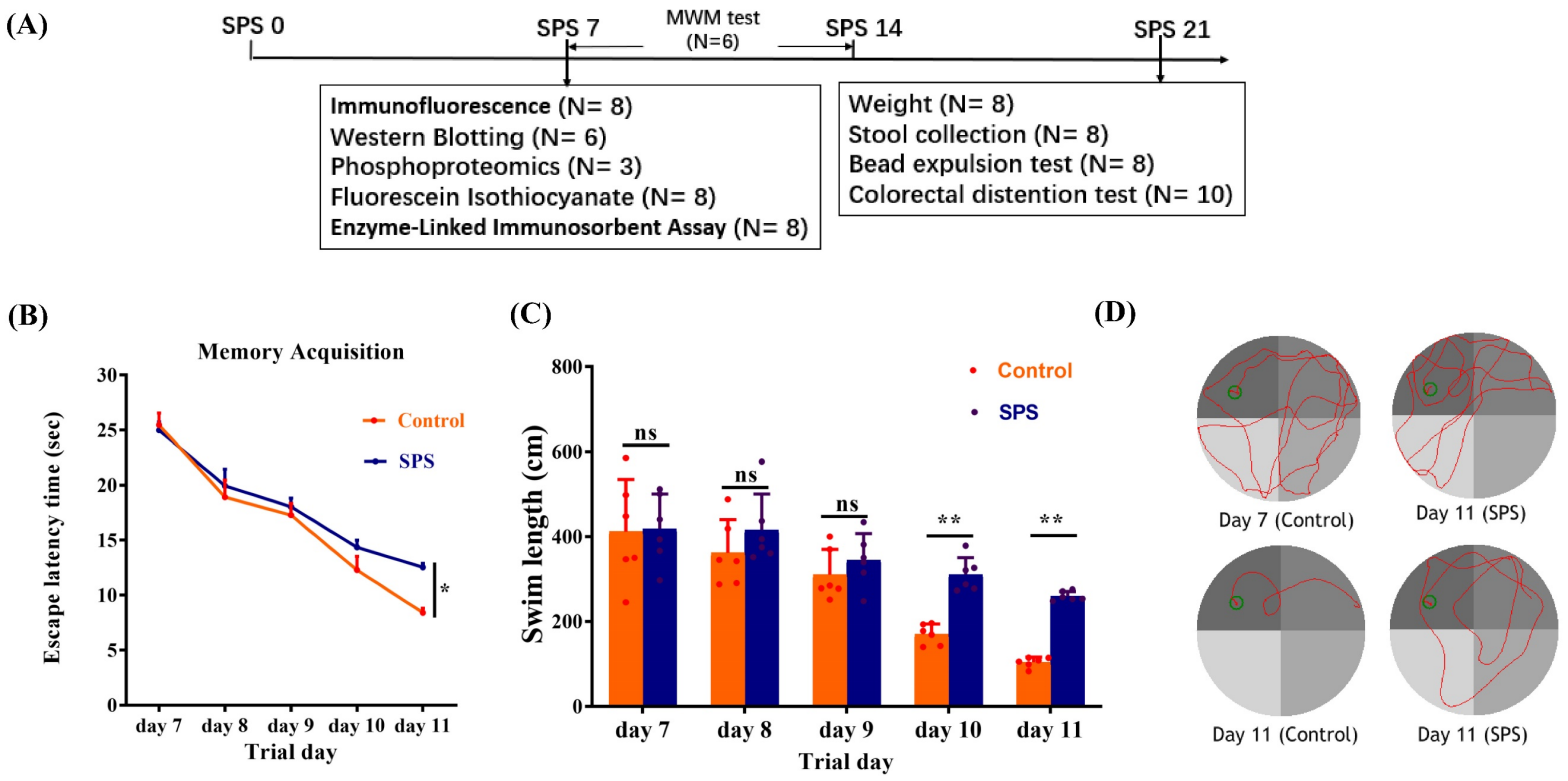
SPS modeling and results of Morris water maze experiment. (A) Overview of the experimental design in mice. (B) Scatter plot illustrating the escape latency of place exploring test. (C) A scatter plot illustrates the swim length of the place exploring test. (D) In the Morris water maze test, the trajectory diagram of control and SPS groups at day 7 and day 11. All data are expressed as the mean ± SEM. The statistical differences were determined using Two-way ANOVA followed by Sidak's multiple comparison test. **p < 0.005, *p < 0.05 compared with control group.

The treatment scheme is illustrated in Figure 1A. In MWM, the escape latency of the SPS group was increased compared to the control group (Figure 1B, *p* = 0.0298). The escape latency for mice in both groups gradually declined from day 1 to day 5. Additionally, the SPS mice exhibited a greater swimming distance than the control mice in the concealed platform assessment on days 10 and 11 (Figure 1C,* p* = 0.003 and *p* = 0.001). These results demonstrate that SPS exposure causes impaired spatial learning and memory.

### SPS exposure affects weight gain and visceral pain

**Figure 2:**
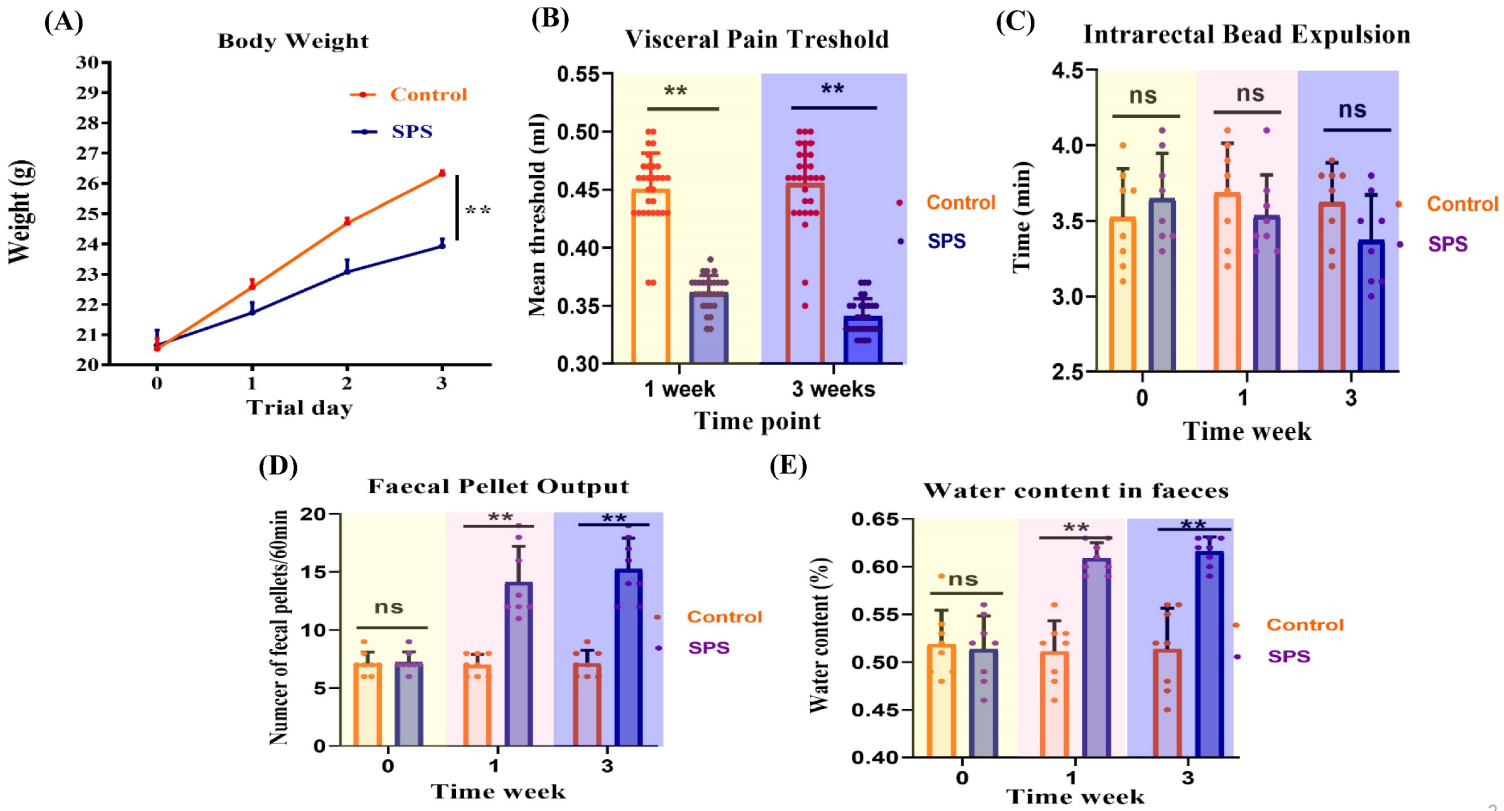
Effect of stress on behavioral changes and assessment of visceral hypersensitivity of mice. (A) Body weight changes of SPS and control mice over the whole process. (B) Scatter plot illustrating the visceral sensitivity of SPS and control mice. Data are expressed as mean ± SEM. Effect of stress on the diarrhea of IBS-like symptoms in mice. (C) Scatter plot illustrating the number of fecal pellet outputs of SPS and control. (D) The moisture content of the feces in two models. (E)Time of glass bead output. All data are expressed as the mean ± SEM. The statistical differences were determined using Two-way ANOVA followed by Sidak's multiple comparison test. **p < 0.0005, *p < 0.05 compared with control group.

Mice in the SPS group gained weight more slowly than those in the control group (Figure 2A,* p* < 0.0001). Changes in visceral sensitivity were detected, as shown in Figure 2B. The SPS group exhibited a lower pain threshold compared to the control group on days 14 and 21 (*p* < 0.0001 and *p* = 0.002). These findings indicate that we have successfully established models of visceral hypersensitivity in IBS.

### Stress-increased defecation and fecal water content

Feces were collected over a one-hour period to determine their water content. SPS mice produced a significantly higher number of fecal pellets during this hour than the controls (Figure 2C, *p* < 0.0001). Additionally, fecal water content was significantly higher in the SPS group compared to the control group (Figure 2D, *p* < 0.0001). These findings suggest abnormal fecal characteristics associated with IBS-like diarrhea symptoms.

### Colonic bead expulsion no change following SPS exposure

As shown in Figure 2E, there was no significant difference in glass bead output time or the amount of feces between the SPS and control groups (*p* = 0.62). These findings indicate that SPS-induced diarrhea is primarily associated with increased fecal water content and the frequency of fecal pellets, without significantly affecting propulsive colonic motility.

### Differential phosphoprotein profiling between the control and SPS treatment models

**Figure 3:**
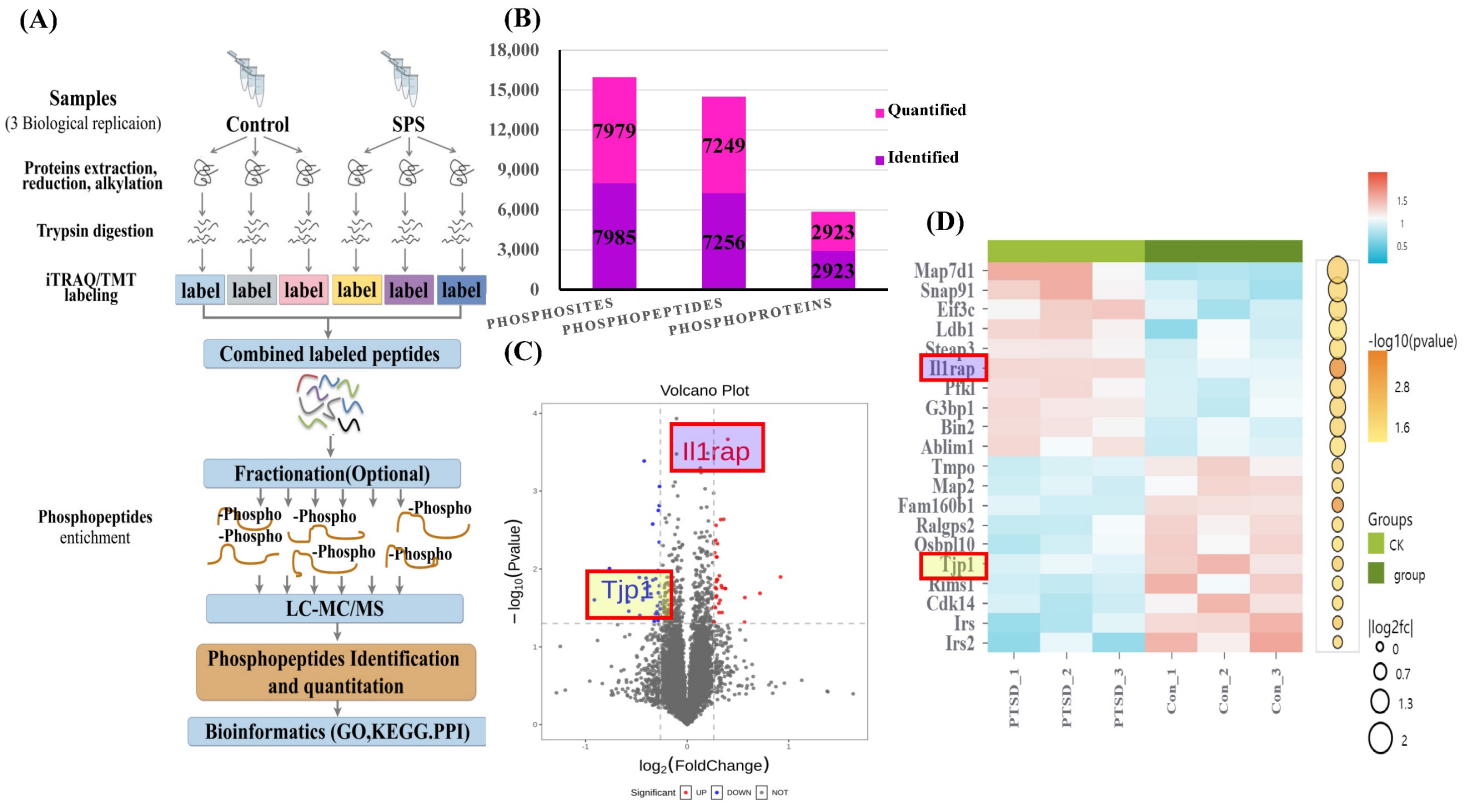
(A) Scheme of Phosphoproteome experimental design. (B) Statistical histogram of identification and quantitative results. (C) Volcano plot of differentially expressed proteins. *X*-axis, Log2FC; *Y*-axis, -Log10 (*P* value). Each point represents an individual protein. The significantly down-regulated modified peptide segments were marked in blue (FC < 0.83 and *p* < 0.05), the up-regulated modified peptide segments were marked in red (FC > 1.2 and *p* < 0.05), and the modified peptide segments without difference were gray. The volcanic map display is applicable to the data obtained by the t-test algorithm. (D) Heatmap showing phosphorylated peptide segments in SPS and control group. Each column represents a group of samples, and each row in the map represents a phosphorylated peptide (i.e., the ordinate is the modified peptide with significant differential expression). The expression of the modified peptide in different samples is standardized by the Z-score method and displayed in different colors in the heat map, in which red represents the significantly up-regulated peptide, and blue represents the significantly down-regulated peptide. White means no peptide quantitative information. Data were from one experiment, and each group contained 3 technological replicates.

The scheme of the phosphoproteome experimental design is illustrated in Figure 3A. In this project, we identified 2923 phosphorylated proteins, 7256 phosphorylated peptides, and 7985 phosphorylated sites. Specifically, we quantified 7249 phosphorylated peptide segments and 7979 phosphorylated sites on these 2923 phosphorylated proteins (Figure 3B).

### Variance result quantity statistics, subcellular localization, cluster and domain analysis

To highlight the significant difference in phosphorylated modified peptide segments between the two groups, we selected phosphorylated peptide segments based on two criteria: fold change (FC) in expression difference and P-value from a t-test (Figure 3C, Table S1 and S2). We analyzed the expression patterns of samples from the control and SPS treatment groups. The phosphorylated peptides with varying expression levels were organized and displayed in a heatmap. Clustering and grouping were performed based on the similarity of results, revealing a high degree of pattern consistency within each group. In contrast, the similarity of data patterns between the two groups was low, indicating that it can successfully distinguish between groups (Figure 3D).

### Classification of Phosphoproteins into Functional Groups

**Figure 4:**
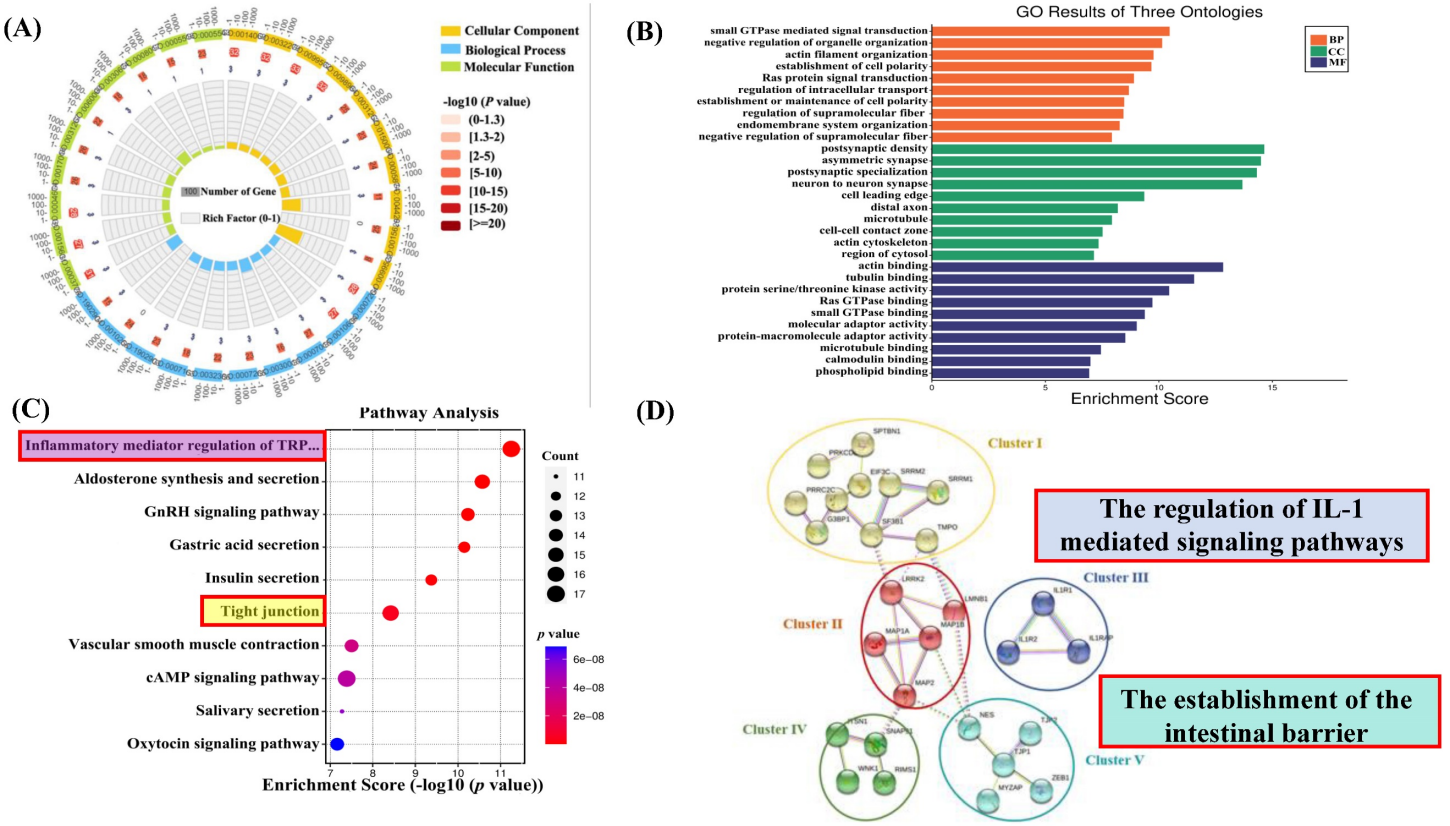
SPS and control group enrichment analysis. (A) GO enrichment circle diagram. The first circle: the GOterm of the first 10 enrichment cycles, and the number of genes outside the circle is a sitting scale. Different colors represent different ontologies; the second circle: the number of GOterm and Q value of the GOterm in the background gene. The more genes, the longer the bar, the smaller the Q value and the redder the color; The third circle: the number of foreground genes under this pathway, and displays them in light purple; the fourth circle: the richfactor value of each GOterm (the number of differences in the GOterm divided by all the quantities), background grid line, each grid represents 0.1). (B) Go enrichment analysis histogram. The closer the color is to red, the smaller the *p*-value. The higher the significance level of the enrichment under the corresponding biological process. Molecular functions and biological process cellular component. The ordinate represents the statistical results of differentially modified proteins under each domain classification, in which the bubble color. The color indicates the significance of the enriched domain classification. That is, the *p*-value is calculated based on the FET. The color gradient represents the size of the *p*-value (take -log10). (C) KEGG pathway analysis of the differentially expressed phosphoproteins. (SPS *vs*. control group, one-sample t-test. The top 20 KEGG pathways are listed (FDR < 0.05). The *X*-axis indicates the corresponding FDR (log10 scale), with the related KEGG pathway marked on the *Y*-axis. The ordinate represents the statistical results of differentially modified proteins under each domain classification, in which the bubble color. The color indicates the significance of the enriched domain classification. That is, the *p*-value is calculated based on the FET. The color gradient represents the size of the *p*-value (take -log10). The closer the color is to red, the smaller the *P* value. (D) Protein-protein interaction network analysis. The interaction network was generated by STRING v10.5 based on the input of 27 differentially expressed phosphoproteins (fold change > 1.2 or <0.83, SPS *vs*. control group, *p* < 0.05, one-sample t-test). The minimum required interaction score was set at medium confidence. The strength of the PPI is indicated by the thickness of the lines in between, and the disconnected nodes in the network are hidden. The subnetworks were clustered by using a K-means algorithm.

To better understand the biological involvement of these phosphoproteins in the process of SPS-induced IBS-like symptoms, a Gene Ontology (GO) examination with protein analysis through evolutionary relationships (PANTHER) system of classification was used to investigate the biological procedure, cellular constituents and molecular functions of these differentially phosphorylated proteins. All differentially expressed proteins' modified peptides were compared with reference species proteins. The significance of the differences between the two groups was determined using Fisher's Exact Test (FET), and the functional classifications of all DEPs were determined (*p* < 0.05). A circle diagram and histogram were used to show the enrichment under the three categories of GO. As shown in Figures 4A and B, significant changes were found in critical biological processes, including postsynaptic density asymmetric synapses. Significant changes were also demonstrated in small GTPase-mediated signal transduction, negative regulation of organelle organization, actin binding, and tubulin binding.

### KEGG Pathway Analysis for Functional Enrichment

To further investigate the biological functions of newly identified signaling and phosphoproteins processes in response to SPS exposure, we analyzed the enrichment of pathways in the KEGG database (comparing SPS vs. control using FET; -log 10 [*p*-value]). In this analysis, a lower *p*-value indicates a higher enrichment of DEPs in a particular pathway (see Figure 4C). The analysis showed that the SPS-induced DEPs were predominantly enriched in several pathways, such as tight junctions (see Figure S1) and inflammatory mediator regulation of TRP channels (see Figure S2). Notably, tight junction phosphorylation was a significant finding.

### STRING Protein-protein Analysis of Differentially Expressed Proteins

The Search Tool for the Retrieval of Interacting Genes (STRING) is a database software for protein-protein interactions. It generates a chain of connections from various sources, including multiple databases of interactions, hereditary interactions, text mining, and standard route connections. This study provides valuable insights into the systemic importance of cellular activities within a functioning biological system (assuming DRG is a specific term for your study's context). Additionally, connections formed by binding proteins reveal possible explanations for how stress impacts colon functioning. In this study, STRING analysis identified significant connections among 27 remarkably regulated phosphoproteins in the control and SPS treatment groups (Figure 4D).

It's worth noting that the PPI network can be divided into five highly connected subnetworks. Cluster I is involved in the regulation of RNA binding (GO:0003723). Cluster II contains phosphoproteins belonging to structural molecule activity (GO:0005198). Cluster III comprises the regulation of interleukin-1-mediated signaling pathways (GO:2000659), including proteins like Interleukin 1 Receptor type I (IL-1RI), Interleukin 1 Receptor type 2 (IL-1R2), and Interleukin-1 receptor accessory proteins (IL-1RAP). Cluster IV includes the synaptic vesicle cycle (GO:0099504). Cluster V comprises a series of processes involved in the positive regulation of blood-brain barrier permeability (GO:1905605) and the establishment of the endothelial intestinal barrier (GO:0090557), such as Tight junction protein ZO-1 (TJP1) and Tight junction protein ZO-2 (TJP2), which may play essential roles in barrier function (assuming BGA refers to barrier function or a similar concept).

### Validation of Identified Differentially Expressed Proteins

**Figure 5:**
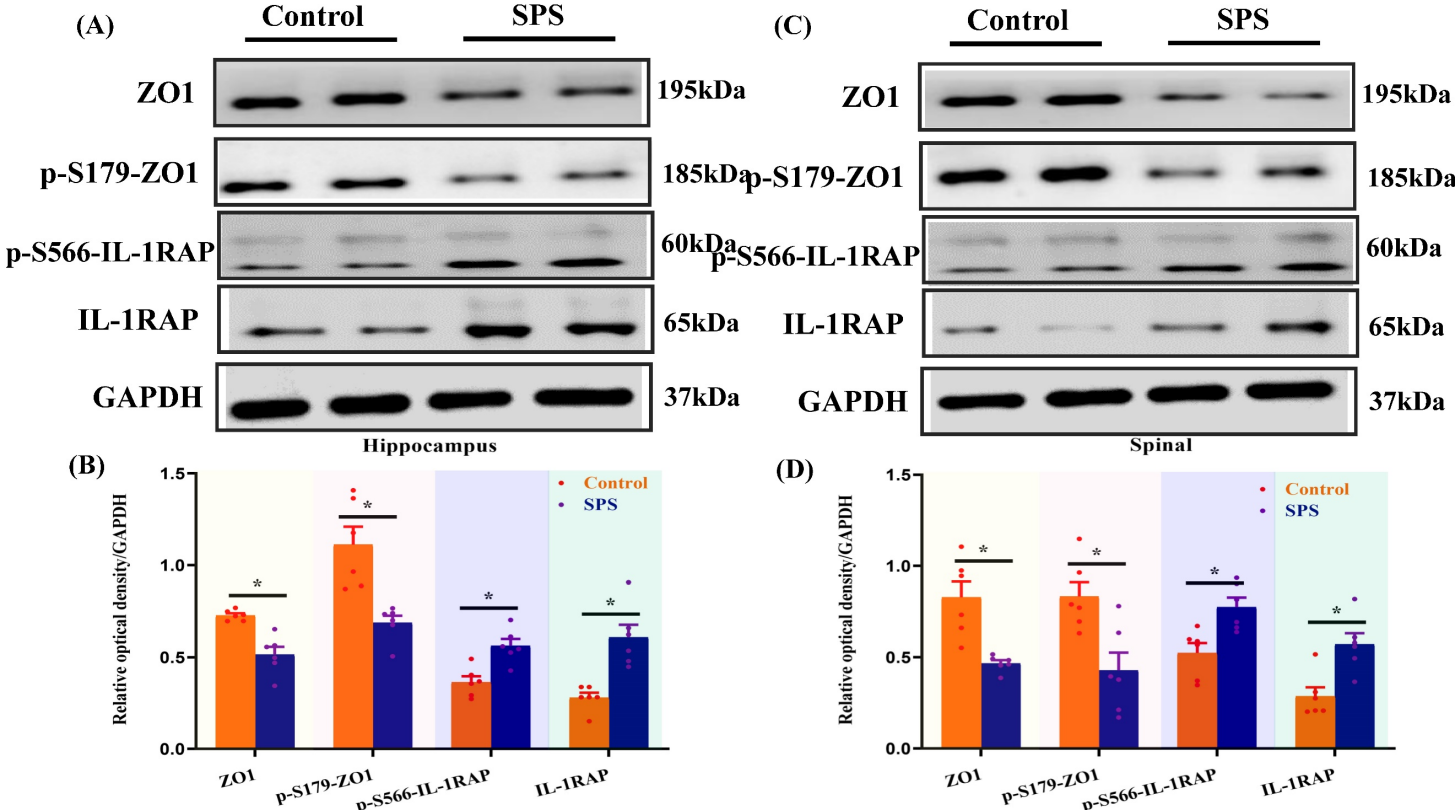

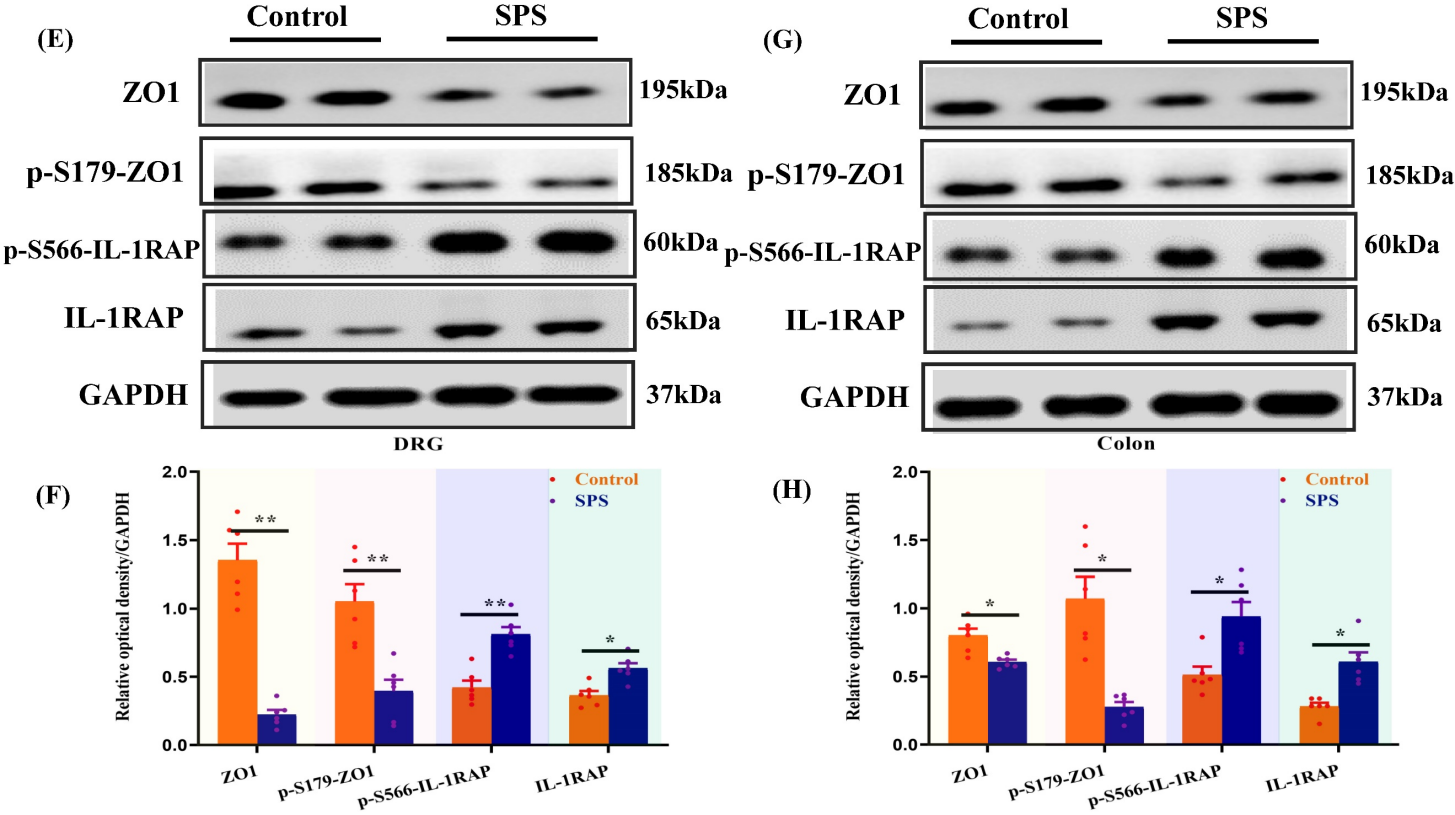

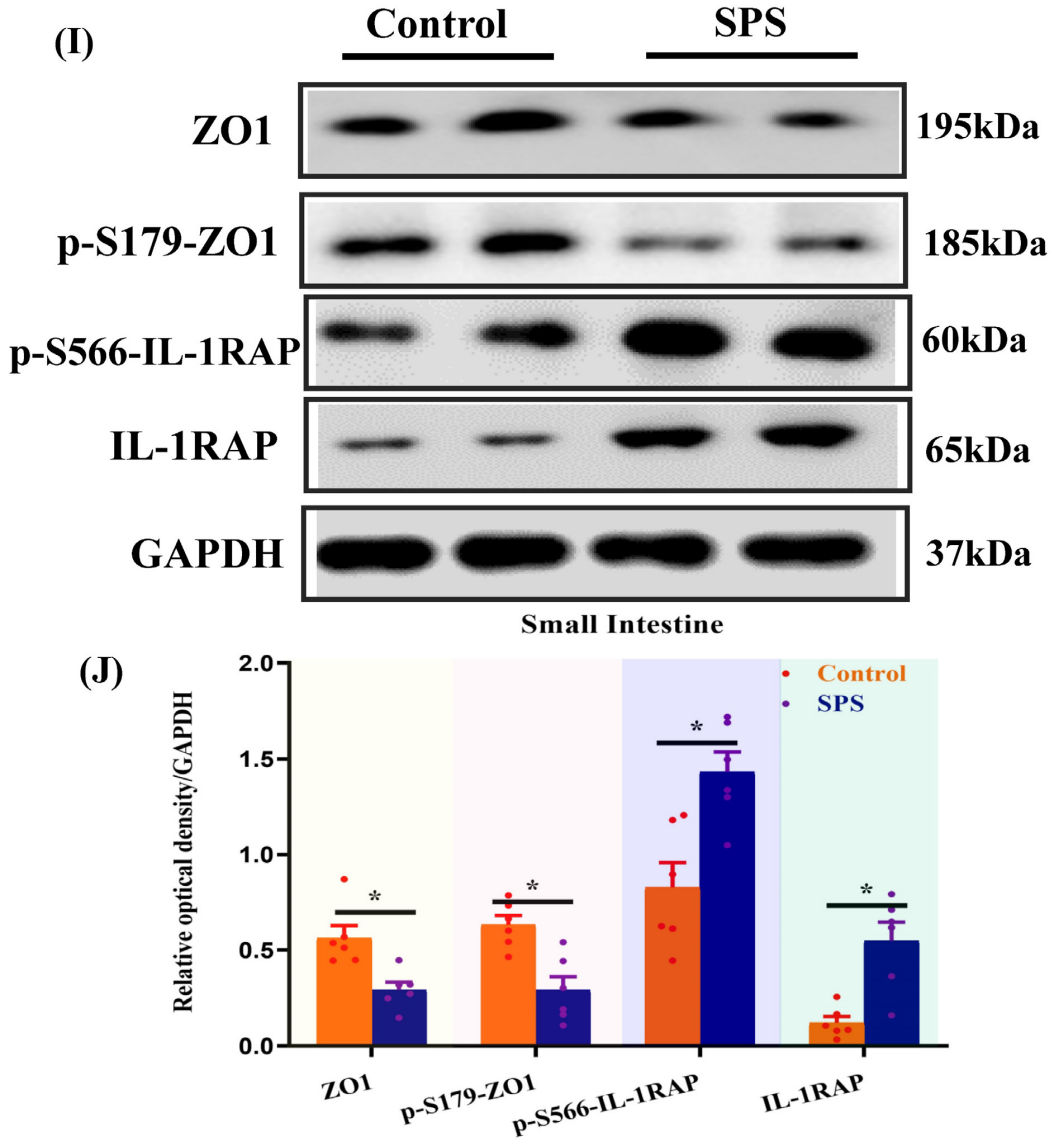
Altered phosphorylation of ZO1 and IL-1RAP was examined by Western blotting. (A) The expression of ZO1, p-S179-ZO1, p-S566-IL-1RAP and IL-1RAP in the hippocampus. (B) The relative optical density of ZO1, p-S179-ZO1, p-S566-IL-1RAP and IL-1RAP expression in the brain of mice following exposure to single-prolonged stress (SPS) as determined by Western blotting. (C) In spinal. (D) The relative optical density of ZO1, p-S179-ZO1, p-S566-IL-1RAP and IL-1RAP expression in the spinal of mice following exposure to SPS as determined by Western blotting. (E) In the dorsal root ganglion (DRG). (F) The relative optical density of ZO1, p-S179-ZO1, p-S566-IL-1RAP and IL-1RAP expression in the DRG of mice following exposure to SPS as determined by Western blotting. (G) In the small intestine. (H) The relative optical density of ZO1, p-S179-ZO1, p-S566-IL-1RAP and IL-1RAP expression in the small intestine of mice following exposure to SPS as determined by Western blotting. (I) In colon. (J) Relative optical density of ZO1, p-S179-ZO1, p-S566-IL-1RAP and IL-1RAP expression in the colon of mice following exposure to SPS as determined by Western blotting. With GAPDH as the loading control. The statistical differences were determined using Two-way ANOVA followed by Sidak's multiple comparison test. **p < 0.005, *p < 0.05 compared with control group.

Phosphopeptides mapped to ZO1 and IL-1RAP displayed significant fold variations in SPS mice (Table S1 and Table S2). The phosphorylation of ZO1 and IL-1RAP was then investigated by Western blot analysis. We observed a significant decrease in the ZO1 phosphorylation in the spinal cord and hippocampus of SPS mice compared to the control group (Figure 5 A,B,C,D) (*p* < 0.05). In line with previous research, we also noted a decrease in ZO1 phosphorylation in DRG, colon, and small intestine (Figure 5 E,F,G,H,I,J) (*p* < 0.05). Consistent with the quantitative phosphoproteomics results, we observed a remarkable increase in phosphorylated IL-1RAP in SPS mice. Meanwhile, the total IL-1RAP protein level was also changed in DRG, colon and small intestine, spinal, and hippocampus.

### Stress-induced Expression Change of ZO1, p- ZO1 and Astrocytes

**Figure 6:**
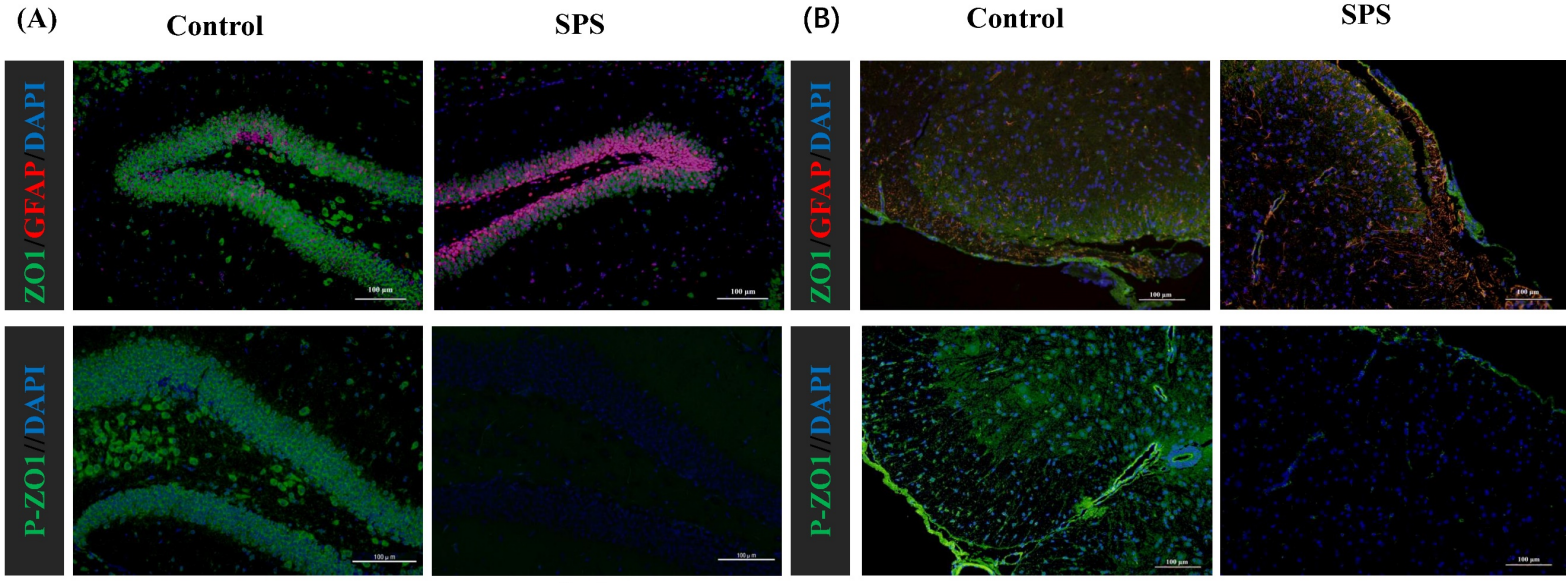

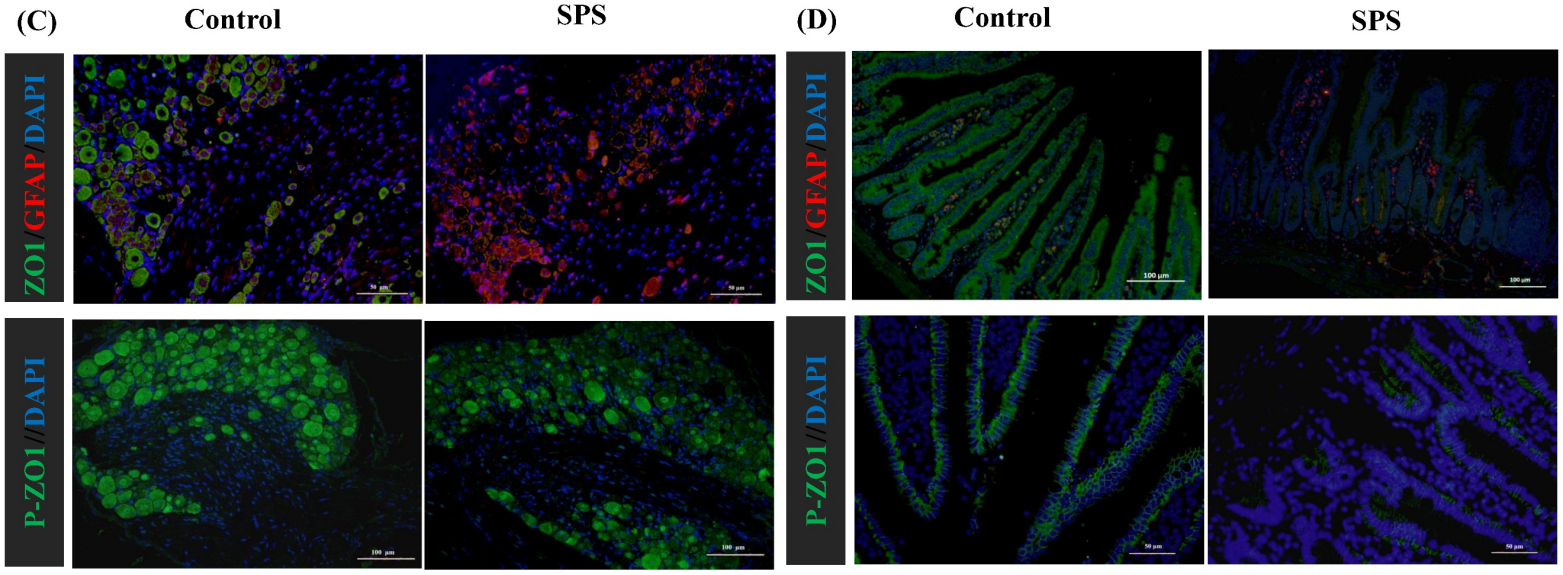

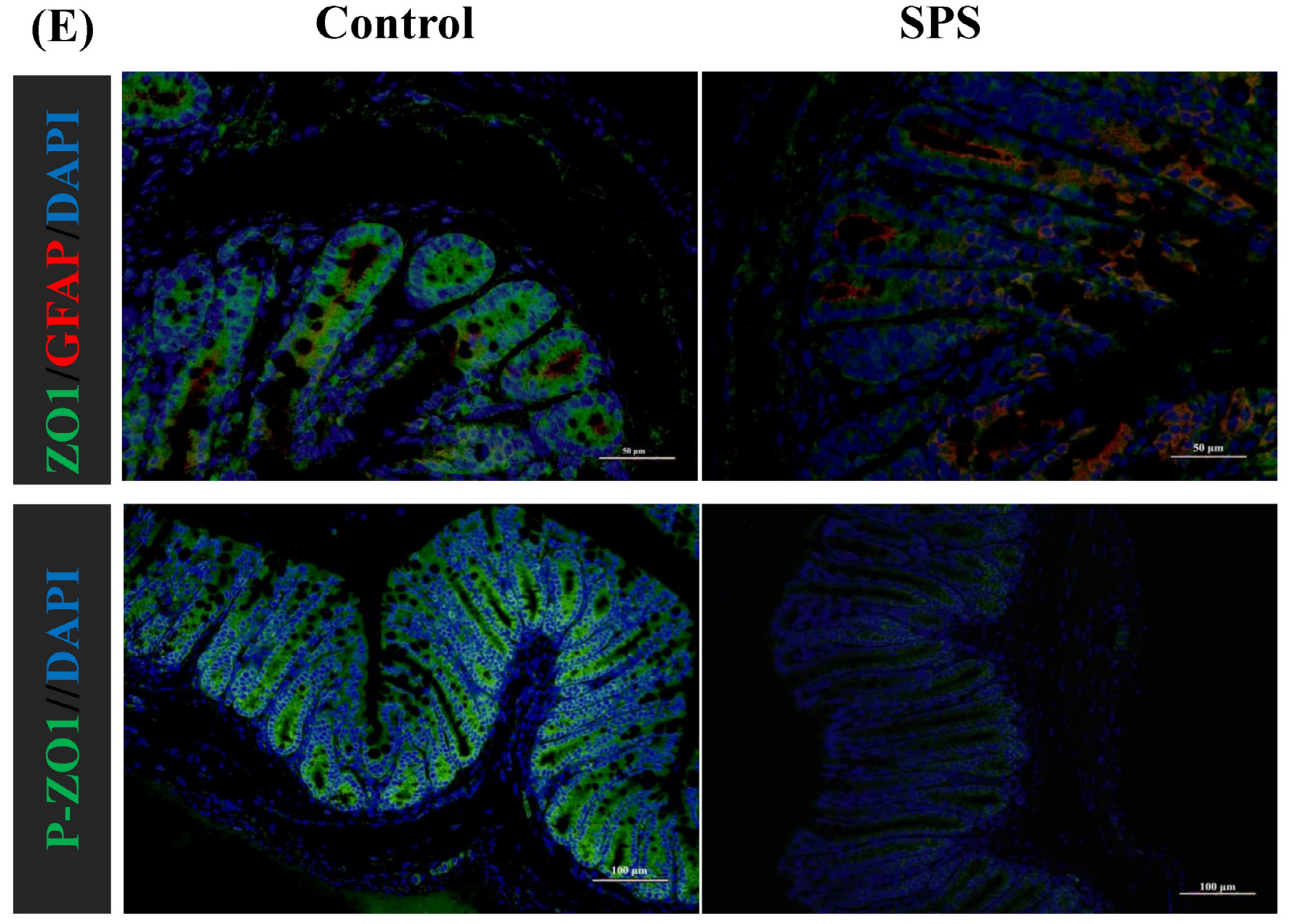
Altered phosphorylation of ZO1 and GFAP was examined by immunofluorescence staining. (A)Representative immunofluorescence images of the ZO1, p-S179-ZO1 and glial cells (GFAP) expression in the hippocampus tissue. (B)In the spinal. (C)In the DRG. (D)In the small intestine. (E) In the colon tissue. Nuclei were stained with 4,6-diamidino-2-phenylindole (DAPI) (blue staining), ZO1 (green staining), p-S179-ZO1 (green staining) and GFAP (red staining).

Immunofluorescence staining was utilized to assess the impact of stress on the phosphorylation status of ZO1, which was linked to alterations in the distribution of the BGA and the 'Neuronal-Glial-Epithelial Unit'. For the BGA, the fluorescence intensity of ZO1 and p-ZO1 protein was decreased in the SPS group compared to the control group in the DRG, spinal and hippocampus (Figure 6 A,B,C). For the 'Neuronal-Glial-Epithelial unit', the fluorescence intensity of ZO1 and p-ZO1 protein was clearly diminished in the SPS group as compared to control in specifically stained intestinal and colon epithelial cells (Figure 6 D,E). Concurrently, a decrease in ZO1 expression and an increase in GFAP expression were observed, indicating an inverse relationship. Therefore, both iTRAQ- and immunoblot-based methods yield comparable results in terms of relative phosphorylation measurement and site recognition. Hence, the results of targeted phosphoprotein analysis were reliable, and alterations in phosphorylation are significantly associated with biological changes.

### The Importance of BGA In the Development of Stress-induced IBS-like Symptoms by IL-1β

**Figure 7:**
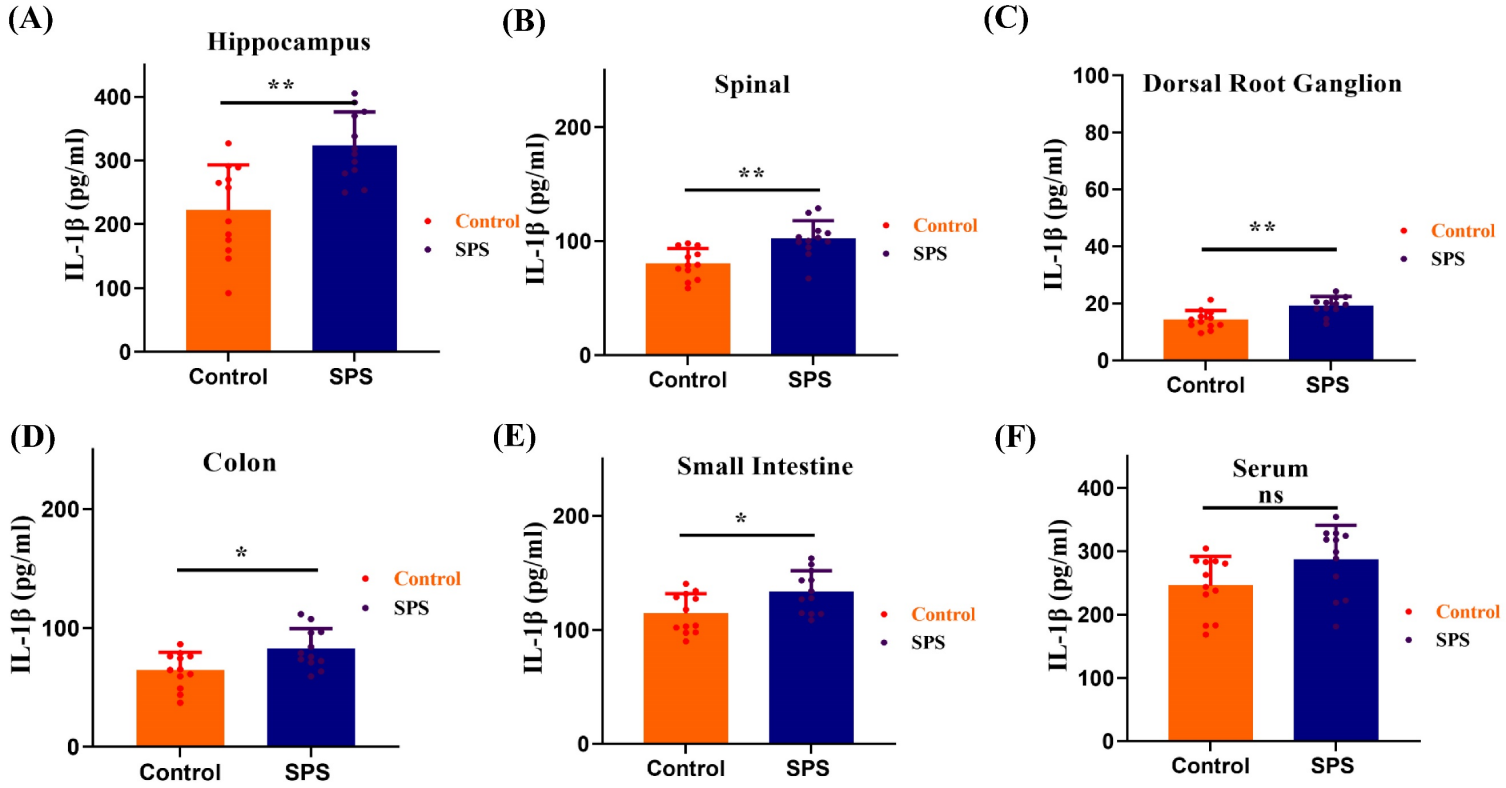
ELISA measured the IL-1β protein levels. (A) The IL-1β protein levels in hippocampus samples. (B) The IL-1β protein levels in spinal samples. (C) The IL-1β protein levels in DRG samples. (D) The IL-1β protein levels in colon samples. (E) The IL-1β protein levels in small intestines samples. (F) The IL-1β protein levels in serum samples. All data are expressed as the mean ± SEM. The statistical differences were determined using the two-tailed t test followed by the Shapiro-Wilk or Kolmogorov-Smirnov test. **p < 0.0005, *p < 0.05 compared with control group.

We observed a significant elevation of IL-1β levels in the spinal cord and hippocampus of SPS mice compared to the control group (Figure 7 A,B, p<0.001). Meanwhile, IL-1β levels also increased in the DRG, colon, and small intestine (Figure 7 C,D,E, *p* < 0.05). However, there was no statistically significant difference in serum IL-1β levels between the SPS and control groups (Figure 7 F, *F* = 1.361, *p* = 0.0583). These findings suggest that the inflammatory response in stressed mice predominantly manifests in the gut-vagus nerve-brain pathway of the BGA, and it does not exert its effects through the humoral circulatory regulatory mechanism within the BGA.

### Alterations in the IEB structure and function of Stress-induced IBS-like Symptoms

**Figure 8:**
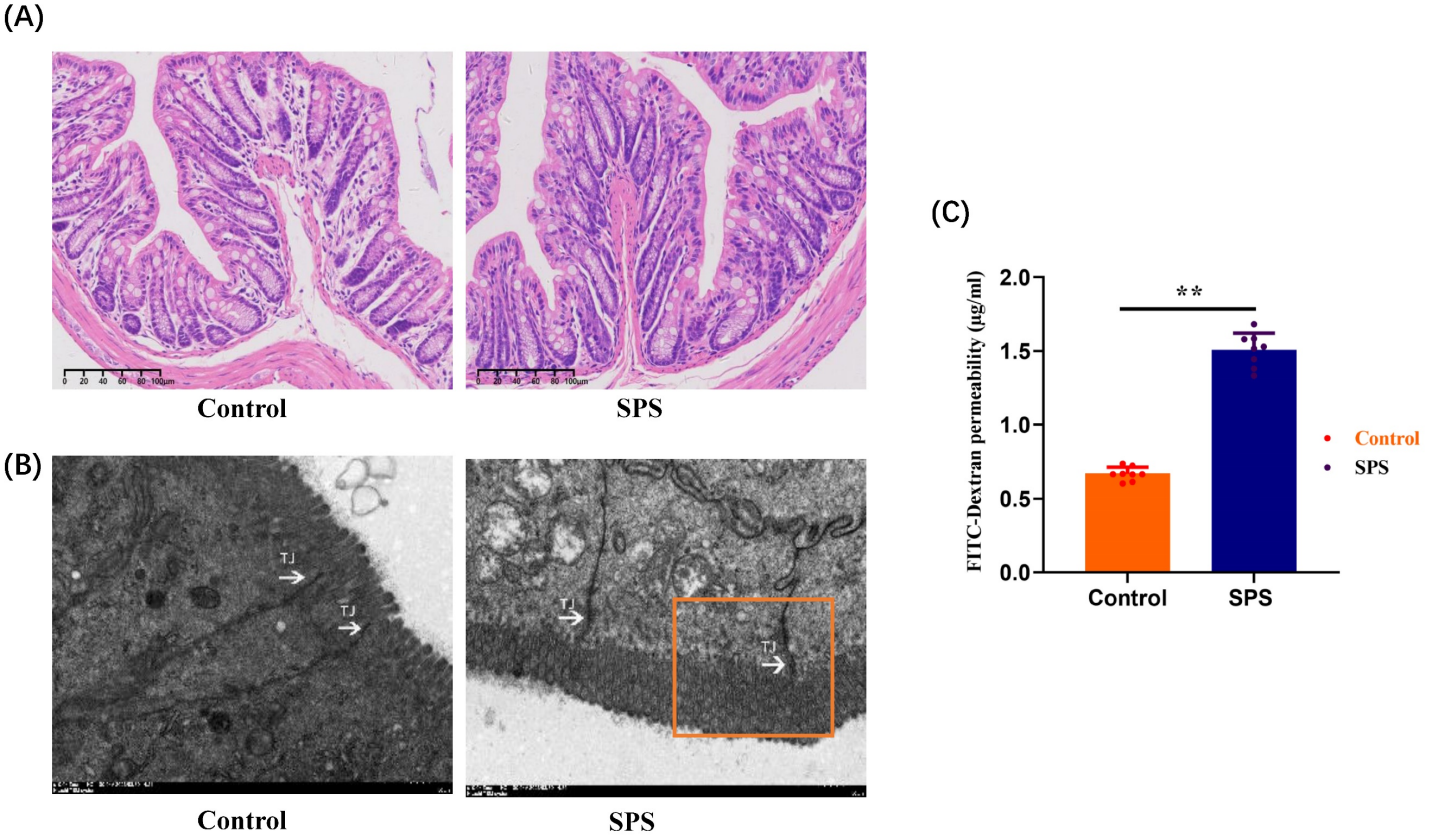
Stress-promoted IEB dysfunction and tight junction barrier loss. (A) H&E-stained colon sections 7 days after SPS; scale bar 100 μm. (B) Transmission electron micrographs of tight junction (TJ) regions in colon enterocytes. Arrow: TJs; scale bar 1 μm. (C) Intestinal permeability assay. All data are expressed as the mean ± SEM. The statistical differences were determined using a two-tailed t-test followed by the Shapiro-Wilk test or Kolmogorov-Smirnov test. **p < 0.0001 compared with the control group.

To distinguish between IBS and inflammatory bowel disease models, we took colon tissues from two groups of mice for H&E staining histopathological analysis. The results showed that the colonic mucosal integrity of mice in both the SPS model group and the control group was good, with no obvious ulcers or signs of mucosal structural integrity loss, and no significant pathological changes were observed in the colon tissue (Figure 8 A). We can effectively distinguish this model from inflammatory bowel disease models based on pathological observation and analysis.

The disruption of tight junction proteins (TJs) plays a significant role in epithelial dysfunction. Based on the close correlation between the phosphorylation of ZO-1 and stress-induced IBS-like symptoms, we observed the structural changes of the IEB in mice using transmission electron microscopy (TEM). Compared to the control group, SPS mice exhibited an enlarged tight junction gap between intestinal epithelial cells and damage to some junctional zones. This indicates that under SPS conditions, morphological changes in the IEB and damage to TJs structures can occur in IBS-like stressed mice (Figure 8 B). To assess whether the phosphorylation of ZO1 alterations in IEB function was associated with intestinal permeability, we identified the concentration of FITC-Dextran 4000 in plasma, reflecting intestinal permeability and critical for IEB. The amount of FITC-Dextran 4000 (1.507±0.041 μg/mL) in plasma was higher in SPS mice than in controls (0.667±0.016 μg/mL) (Figure 8 C, *F* = 6.255,* p* < 0.0001). This implies that the phosphorylation level of ZO-1 is crucial for maintaining the integrity and function of the intestinal barrier structure.

## Discussion

In this study, after treatment of SPS, the Morris Water Maze (MWM) test revealed deteriorations in learning and spatial memory 46, 47. Our results align with previous studies indicating that the SPS model is often associated with hippocampal-dependent memory impairments, which is a significant symptom cluster in PTSD. The AWR test assessed the changes in visceral pain thresholds following SPS exposure. The outcomes of visceral hypersensitivity-related experiments suggested that SPS exposure caused visceral pain of IBS-like Syndrome in the SPS-treated mice. The exaggerated stress response observed in the SPS model may contribute to increased visceral hypersensitivity, which may predispose individuals to the development of IBS. Following SPS exposure, the number of fecal particles and fecal water content was considerably higher, which is adequate to induce signs of diarrhea. At the same time, we observed less weight gain during the SPS. Therefore, The SPS model induced changes in gastrointestinal physiology, including visceral hypersensitivity, reduced weight gain, and diarrhea-like symptoms, which overlap symptoms with inflammatory bowel disease and IBS. In contrast, no gross lesions and inflammatory mucosa existed between SPS-treated and control mice. Based on pathological observations and analyses, we can clearly distinguish this model from models of inflammatory bowel disease. Based on these findings, we conclude that SPS-treated mice exhibit visceral hypersensitivity and diarrhea symptoms characteristic of IBS. TEM revealed that the tight junctions of colonic epithelial cells were damaged, which may lead to the destruction of the intestinal mucosal barrier structure. Meanwhile, FITC-D4000 experiments functionally verified intestinal epithelial dysfunction and the increase of intestinal mucosal barrier permeability.

This study assessed relative alterations in protein phosphorylation of DRG neurons to explore the epigenetic mechanism of visceral hypersensitivity in IBS-like stressed mice. Our finding identified a slew of phosphoproteins with numerous potential phosphorylation sites from three dimensions. GO enrichment analysis indicated that differentially phosphorylated proteins regulate tight junctions and maintain blood-brain barrier (BBB) permeability. Cluster analysis revealed those significant differences in interleukin-1-mediated signaling pathway (GO:2000659), positive regulation of blood-brain barrier permeability (GO:1905605) and Establishment of endothelial intestinal barrier (GO:0090557). Moreover, their phosphorylation sites may play essential roles in BGA. As for KEGG pathways, the enrichment results showed that tight junction, and inflammatory mediator regulation of TRP channels were enriched in phosphoproteome. The results indicated significant differential expression of p-S179-ZO1 and p-S566-IL-1RAP between SPS-treated and control mice. These two phosphorylated proteins could regulate multiple molecules, such as epithelial barrier dysfunction and BBB permeability through BGA and inflammatory mediator regulation of TRP channels. They were identified as the potential pathologic “checkpoints” of the BGA. Additionally, we measured the protein expression of ZO1, p-S179-ZO1, IL-1RAP, and p-S566-IL-1RAP in mice's intestinal, colon DRG, spinal and hippocampus tissues by immunofluorescence and western blotting to further verify our bioinformatics analysis results.

According to the Rome IV Guidelines of Functional gastrointestinal abnormalities, IBS is a BGA medical problem, emphasizing the significance of the interactions between intestinal, memory, and emotional symptoms in IBS 48. The clinical symptoms of IBS typically include abdominal pain and diarrhea, and one challenge in diagnosing IBS is the lack of specific biomarkers 3. Growing studies have indicated different mechanisms in the pathogenesis of IBS, including visceral hypersensitivity, intestinal barrier dysfunction 4, intestinal inflammation 49, and imbalance of intestinal flora 50. All these factors contribute significantly to the pathogenesis of IBS 51. However, there is still a significant gap in our understanding of how these various factors interact and contribute to the pathogenesis of IBS.

There are two barriers to BGA: the BBB and the intestinal barrier 7. Both of the obstacles are dynamic. Inflammatory signals, stress, and gut microbiota can modulate their permeability 52. TJs, critical components of epithelial cell structure, play a crucial role in junction formation, barrier control, gene transcription, cell polarity, and other processes, particularly at the IEB and BBB 53-55. Inflammation and stress have been shown to disrupt TJs, which is a key factor in abnormal intestinal permeability in IBS and BBB integrity dysfunction in depression 56, 57. TJs consist of many protein aggregates that form a porous, specific bridge between neighboring epithelial cells. Studies have reported a reduction in ZO-1 expression in IBS with diarrhea (IBS-D), while changes in Claudin1 and Occludin expression levels were minimal 58. This finding suggests that alterations in ZO-1 expression may contribute to the intestinal permeability changes seen in IBS-D. Interestingly, in our study, ZO-1 was observed to have a low degree of post-translational modifications following SPS exposure, suggesting that phosphorylation of ZO-1 may be a “key point” connected with IEB and BGA to regulate the stress-induced IBS-like symptoms. To our knowledge, this is the first time the role of phosphorylation at the ZO-1 site on S179 has been implicated in the pathological process of visceral hypersensitivity.

Accumulating experimental and clinical studies have reported that disturbances in both the peripheral and central nervous systems and cytokines can significantly impact gastrointestinal function 59. However, the precise crosstalk mechanism between the BGA and the IEB through inflammatory cytokines in IBS remains elusive. Cytokines, particularly IL-1β, have been shown to penetrate the BBB and interact with neurons, astrocytes, and microglia 60. High concentrations of IL-1β are found in the hippocampus, a region crucial for memory formation 61. Additionally, IL-1β may contribute to various adverse cognitive effects, which can be mitigated by melatonin in cases of sleep disorders and emotional stress 62. IL-1β is a significant pro-inflammatory factor that may trigger an inflammatory response and modulate BBB permeability and mucosal barrier breakdown 5, 63-65. Because of its direct effects on the mucosa and colonic smooth muscle, IL-1β can induce constriction and inflammatory discomfort 66. Notably, IL-1β has emerged as a reliable predictor of IBS-D in the colon and is strongly associated with indicators of pain and depression. Studies have demonstrated that adenosine triphosphate (ATP) stimulates the release of IL-1β from astrocytes, leading to the breakdown of TJs and compromising the integrity of the BBB following brain trauma or chronic central nervous system stress 60. Consistent with these findings, our results indicate an increase in IL-1β levels, suggesting low-grade inflammation within the BGA.

Genome research has shown that IL-1β binds to IL-1RAP and IL-1RI, two crucial receptors part of a larger receptor superfamily essential for rapid initial immune responses 67. IL-1RAP plays a vital role in signal transmission for the functioning IL-1R and could potentially serve as a new target for inhibiting the effects of IL-1 in various human illnesses 68. Additionally, IL-1RAP has been implicated in behavioral modifications observed during periods of increased inflammation 60, 69. However, it remains unknown whether IL-1RAP plays a role in the pathophysiology of IBS. Our findings suggest that stress conditions lead to elevated levels of pro-inflammatory plasma cytokines, activating IL-1β signaling. This signaling promotes the phosphorylation of IL-1RAP and ZO1 in the BBB, increasing its permeability. As a result, IL-1β can pass through the BBB, potentially mediating the destruction of the intestinal epithelial barrier (IEB) and contributing to visceral hypersensitivity (See Graphical Abstract).

In this study, we demonstrated that: (1) SPS Model Effectiveness: SPS model mimics IBS symptoms under stress, similar to clinical cases; (2) BGA Mechanisms in IBS: Phosphoproteome and bioinformatic methods analysis for the first time reveals p-S179-ZO1 and p-S566-IL-1RAP could be the potential pathologic “checkpoints” in mediated BGA in IBS-like stressed mice; and (3) ZO-1 and IL-1RAP Phosphorylation's Impact: SPS stress alters ZO-1 phosphorylation, disrupting the intestinal barrier, increasing IL-1β release, and promoting phosphorylated IL-1RAP complexes, leading to IBS.

There may be some limitations to this study. Firstly, we acknowledge uncertainty regarding the observed diarrheal and visceral hypersensitivity. However, whether the SPS model more accurately mimics human IBS remains unknown. Secondly, it has not been established whether cytokines, including IL-6, IL-8, and other molecules, such as Toll-like receptor 4, have a comparable effect on the visceral hypersensitivity induced by SPS. This area requires further investigation. Thirdly, our observations suggest that the phosphorylation of ZO-1 and IL-1RAP-mediated permeability may contribute to visceral hypersensitivity. Future research should include *in vivo* and *in vitro* experiments to explore this further. Lastly, considering the predominance of stress-induced visceral hypersensitivity in females, future studies should investigate sex differences in the observed effects.

## Conclusion

This study illuminates the vital role of ZO-1 and IL-1RAP phosphorylation in the pathogenesis of IBS, particularly under stress-induced conditions. Our findings demonstrate that ZO-1 and IL-1RAP phosphorylation alterations contribute significantly to enlarged permeability and visceral hypersensitivity in IBS-like stressed mice. These insights deepen our understanding of the molecular mechanisms of phosphorylation pathways in IBS and underscore the importance of the BGA in its pathology. Taken together, our research results offer new insight into the pathological mechanisms of visceral hypersensitivity and diarrhea of IBS and possible biological markers for sensing initial pathological modifications.

## Supplementary Material

Supplementary figures and tables.

## Abbreviations

IBS: irritable bowel sSyndrome
BGA: brain-gut axis
BBB: blood-brain barrier
MWM: morris water maze
SPS: single prolonged stress
AWR: abdominal withdrawal reflex
IEB: intestinal epithelial barrier
DRG: dorsal root ganglion
MS: mass spectrometry
iTRAQ: isobaric tags for relative and absolute quantitation
PTSD: post-traumatic stress disorder
NS: normal saline
IL-1β: interleukin-1 beta
H&E: hematoxylin & eosin
TMTs: tandem mass tags
DEPs: differentially expressed proteins
KEGG: kyoto encyclopedia of genes and genomes
FET: field effect transistor
PPI: frotein-protein interaction
ODs: optical densities
DAPI: 4',6-diamidino-2-phenylindole
FITC-D4000: fluorescein csothiocyanate-dextran 4000
FC: fold change
TJs: tight junction proteins
ATP: adenosine triphosphate
