# Supplementary material for: ZO-1 and IL-1RAP Phosphorylation: Potential Role in Mediated Brain-Gut Axis Dysregulation in Irritable Bowel Syndrome-like Stressed Mice
Source: Int J Med Sci. 2024 Jul 2;21(9):1738–55. doi: 10.7150/ijms.95848 (PMC11241095; doi:10.7150/ijms.95848)
Supplement: Supplementary file 1 — Supplementary figures and tables. [file ijmsv21p1738s1.zip › Supplementary Materials/Supplementary Materials all.docx]

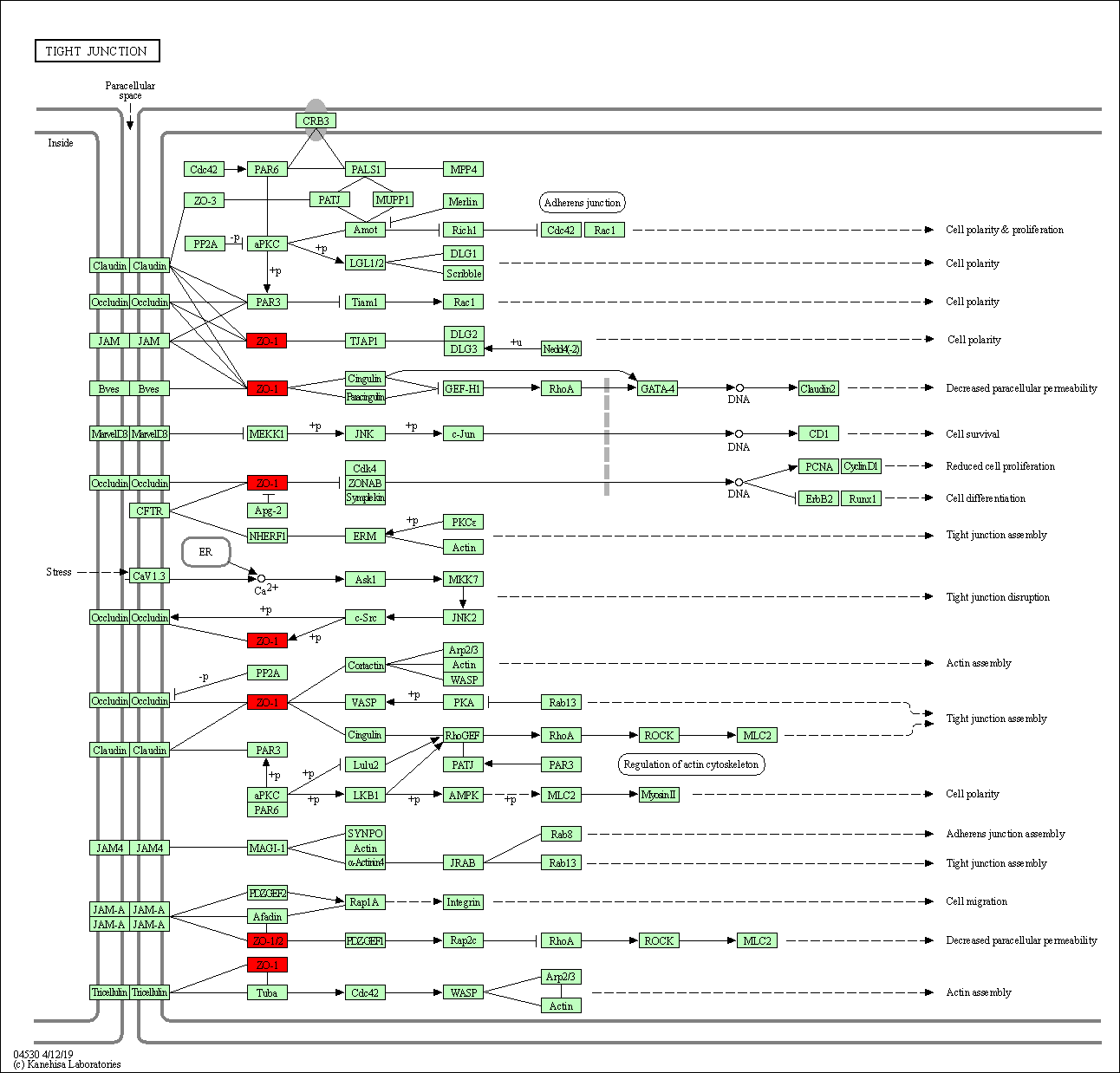


**Figure S1.** Tight junction (modified from reference pathway mmu04530 in KEGG database). The small circle represents small molecule metabolites, the large circle represents other pathways, the light green bottom box is species-specific protein, and the red font is KEGG default disease-related protein.


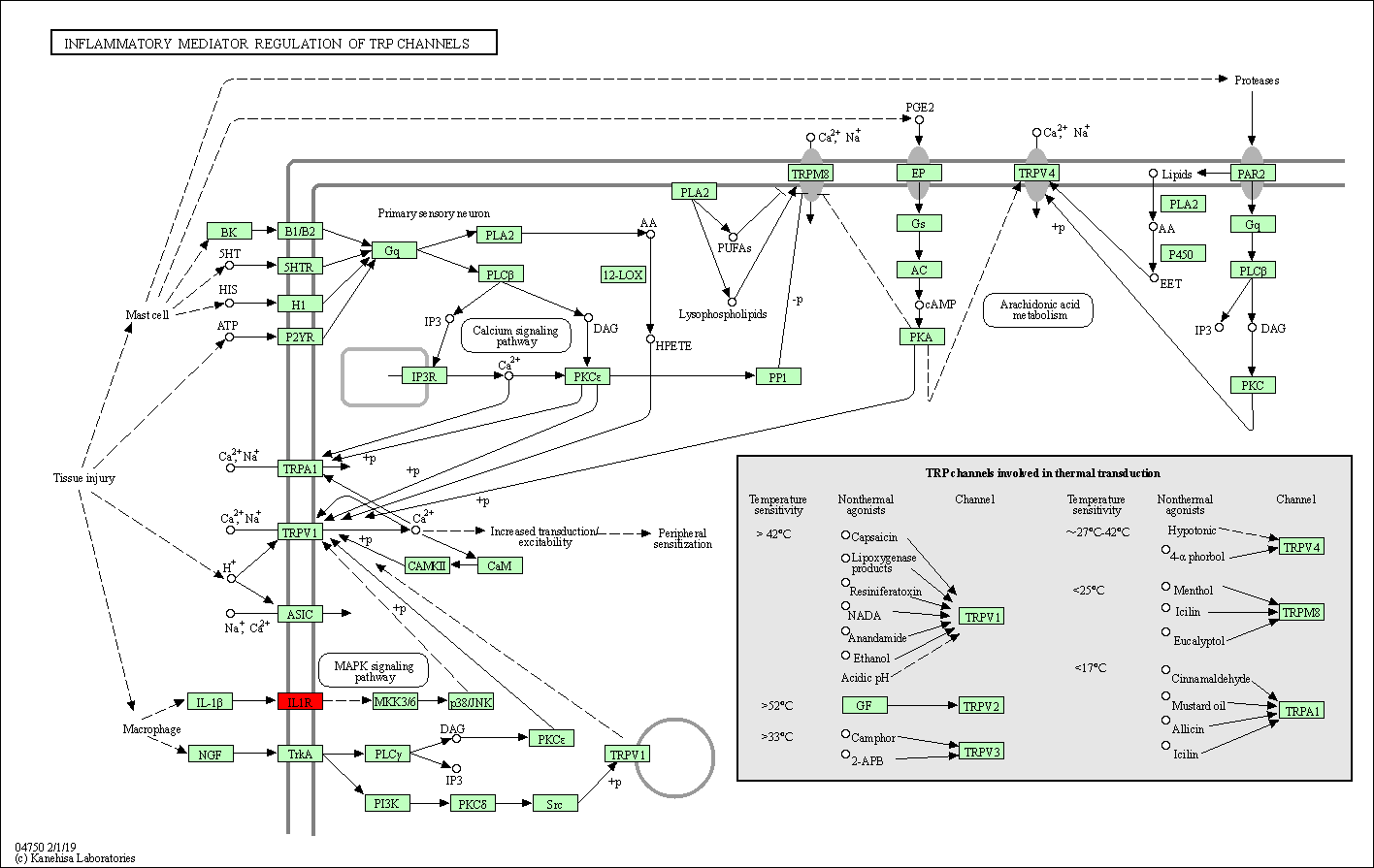


**Figure S2.** Inflammatory mediator regulation of TRP channels (modified from reference pathway mmu04750 in KEGG database). The small circle represents small molecule metabolites, the large circle represents other pathways, the light green bottom box is species-specific protein, and the red font is KEGG default disease-related protein, and the red box indicates that the modified peptides with differences in the modified proteins are up-regulated.

Table S1. Upregulated Phosphorylated Proteins in DRG of IBS-D group Compared to the control group.

| **Protein Accessions** | **Protein Names** | **Gene Names** | **Modifications in Proteins** | **Fold Change** | **P.Value** |
| --- | --- | --- | --- | --- | --- |
| A2AJI0 | MAP7 domain-containing protein 1 | Map7d1 | A2AJI0 1xPhospho [S118(97.5)] | 1.892269 | 0.012658 |
| Q61548 | Clathrin coat assembly protein AP180 | Snap91 | Q61548 2xPhospho [S306(97.2);] | 1.645428 | 0.020537 |
| Q8R1B4 | Eukaryotic translation initiation factor 3 subunit C | Eif3c | Q8R1B4 1xPhospho [S907(100)] | 1.484486 | 0.02329 |
| P70662 | LIM domain-binding protein 1 | Ldb1 | P70662 1xPhospho [S410(99.7)] | 1.480636 | 0.048049 |
| P12382 | ATP-dependent 6-phosphofructokinase, liver type | Pfkl | P12382 1xPhospho [S775(100)] | 1.299811 | 0.017835 |
| P97855 | Ras GTPase-activating protein-binding protein 1 | G3bp1 | P97855 1xPhospho [T230(96)] | 1.297765 | 0.017869 |
| Q61730 | Interleukin-1 receptor accessory protein | Il1rap | Q61730 1xPhospho [S566(100)] | 1.283638 | 0.002309 |
| D3Z6Q9 | Bridging integrator 2 | Bin2 | D3Z6Q9 1xPhospho [S392(100)] | 1.270258 | 0.017481 |
| Q8K4G5 | Actin-binding LIM protein 1 | Ablim1 | Q8K4G5 1xPhospho [S/T/Y] | 1.269772 | 0.036214 |
| Q8BY87 | Ubiquitin carboxyl-terminal hydrolase 47 | Usp47 | Q8BY87 1xPhospho [S849(100)] | 1.264599 | 9.1E-05 |
| Q9Z0U1 | Tight junction protein ZO-2 | Tjp2 | Q9Z0U1 1xPhospho [S674(100)] | 1.263791 | 0.016392 |
| Q8VDD5 | Myosin-9 | Myh9 | Q8VDD5 1xPhospho [S1943(100)] | 1.258197 | 0.002334 |
| Q8BTI8 | Serine/arginine repetitive matrix protein 2 | Srrm2 | Q8BTI8 2xPhospho [S1058(97.8); S1064(99.9)] | 1.253135 | 0.025231 |
| P12382 | ATP-dependent 6-phosphofructokinase, liver type | Pfkl | P12382 1xPhospho [S775(100)] | 1.246825 | 0.012233 |
| Q8VDD5 | Myosin-9 | Myh9 | Q8VDD5 1xPhospho [S1943(100)] | 1.243633 | 0.036266 |
| Q9Z0R4 | Intersectin-1 | Itsn1 | Q9Z0R4 1xPhospho [S971(100)] | 1.242134 | 0.027709 |
| Q5SWP3 | NAC-alpha domain-containing protein 1 | Nacad | Q5SWP3 1xPhospho [S1158(98.5)] | 1.230831 | 0.013768 |
| Q3TLH4 | Protein PRRC2C | Prrc2c | Q3TLH4 1xPhospho [S899(94.8)] | 1.230724 | 0.004733 |
| Q9WTU0 | Lysine-specific demethylase PHF2 | Phf2 | Q9WTU0 1xPhospho [S1057(99.9)] | 1.226117 | 0.006992 |
| Q91ZB8 | Mas-related G-protein coupled receptor member D | Mrgprd | Q91ZB8 1xPhospho [S292(100)] | 1.224392 | 0.013621 |
| Q99NB9 | Splicing factor 3B subunit 1 | Sf3b1 | Q99NB9 2xPhospho [T244(99.8); T248(99.8)] | 1.222956 | 0.022086 |
| Q8R3V6 | CUE domain-containing protein 1 | Cuedc1 | Q8R3V6 1xPhospho [S10(100)] | 1.22138 | 0.006993 |
| Q9QYB8 | Beta-adducin | Add2 | Q9QYB8 2xPhospho [S618(100); T/S] | 1.221053 | 0.018006 |
| Q8K1S6 | Protein spire homolog 2 | Spire2 | Q8K1S6 1xPhospho [S706(100)] | 1.220939 | 0.014622 |
| Q9QYY0 | GRB2-associated-binding protein 1 | Gab1 | Q9QYY0 1xPhospho [S684(99.9)] | 1.219395 | 0.023046 |
| Q8BGD9 | Eukaryotic translation initiation factor 4B | Eif4b | Q8BGD9 2xPhospho [S497(98); S504(97.9)] | 1.21874 | 0.00276 |
| Q64318 | Zinc finger E-box-binding homeobox 1 | Zeb1 | Q64318 1xPhospho [S684(98.1)] | 1.217087 | 0.00428 |
| Q8K3I9 | Glucocorticoid-induced transcript 1 protein | Glcci1 | Q8K3I9 1xPhospho [S26(97.4)] | 1.213509 | 0.031366 |
| Q80X50 | Ubiquitin-associated protein 2-like | Ubap2l | Q80X50 1xPhospho [S629(100)] | 1.211404 | 0.017307 |
| Q3UW53 | Protein Niban 1 | Niban1 | Q3UW53 1xPhospho [S595(100)] | 1.207664 | 0.006172 |
| P32037 | Solute carrier family 2, facilitated glucose transporter member 3 | Slc2a3 | P32037 1xPhospho [S471(100)] | 1.203764 | 0.04823 |
| Q9WV92 | Band 4.1-like protein 3 | Epb41l3 | Q9WV92 1xPhospho [S601(97.6)] | 1.20371 | 0.030575 |
| Q8CI59 | Metalloreductase STEAP3 | Steap3 | Q8CI59 1xPhospho [S20(99.9)] | 1.200535 | 0.030529 |

Table S2. Downregulated Phosphorylated Proteins in DRG of IBS-D group Compared to the control group.

| **Protein Accessions** | **Protein Names** | **Gene Names** | **Modifications in Proteins** | **Fold Change** | **P.Value** |
| --- | --- | --- | --- | --- | --- |
| P14873 | Microtubule-associated protein 1B | Map1b | P14873 1xPhospho [T340(98.9)] | 0.833 | 0.0326 |
| Q6KAU7 | Pleckstrin homology domain-containing family G member 2 | Plekhg2 | Q6KAU7 1xPhospho [S465(100)] | 0.833 | 0.027 |
| Q9EQW7 | Kinesin-like protein KIF13A | Kif13a | Q9EQW7 1xPhospho [S1633(99.9)] | 0.832 | 0.0394 |
| O35144 | Telomeric repeat-binding factor 2 | Terf2 | O35144 1xPhospho [S399(100)] | 0.829 | 0.0009 |
| P59114 | mRNA (2'-O-methyladenosine-N(6)-)-methyltransferase | Pcif1 | P59114 1xPhospho [S116(100)] | 0.828 | 0.0165 |
| Q91VJ2 | Caveolae-associated protein 3 | Cavin3 | Q91VJ2 1xPhospho [S62(100)] | 0.828 | 0.0015 |
| S4R1M9 | Oxysterol-binding protein-related protein 10 | Osbpl10 | S4R1M9 1xPhospho [S/T] | 0.827 | 0.0045 |
| Q8BXQ8 | Protein FAM53C | Fam53c | Q8BXQ8 1xPhospho [S273(100)] | 0.823 | 0.0203 |
| Q62261 | Spectrin beta chain, non-erythrocytic 1 | Sptbn1 | Q62261 2xPhospho [S2164(97.9); S/T] | 0.822 | 0.0018 |
| Q5S006 | Leucine-rich repeat serine/threonine-protein kinase 2 | Lrrk2 | Q5S006 1xPhospho [S973(97.9)] | 0.821 | 0.0459 |
| P14873 | Microtubule-associated protein 1B | Map1b | P14873 1xPhospho [S1952(100)] | 0.821 | 0.0105 |
| P59808 | SAM and SH3 domain-containing protein 1 | Sash1 | P59808 1xPhospho [S241(100)] | 0.818 | 0.0376 |
| P39447 | Tight junction protein ZO-1 | Tjp1 | P39447 1xPhospho [S179(97.4)] | 0.818 | 0.0328 |
| Q9D2V7 | Coronin-7 | Coro7 | Q9D2V7 1xPhospho [S428(84.6)] | 0.817 | 0.0297 |
| Q6P5H2 | Nestin | Nes | Q6P5H2 1xPhospho [S728(100)]; 1xPhospho [S772(100)] | 0.814 | 0.0354 |
| Q9D823 | 60S ribosomal protein L37 | Rpl37 | Q9D823 1xPhospho [S97(98.1)] | 0.807 | 0.0134 |
| P14733 | Lamin-B1 | Lmnb1 | P14733 1xPhospho [S24(97.9)] | 0.806 | 0.0377 |
| P83741 | Serine/threonine-protein kinase WNK1 | Wnk1 | P83741 1xPhospho [S185(99.9)] | 0.799 | 0.0468 |
| Q52KI8 | Serine/arginine repetitive matrix protein 1 | Srrm1 | Q52KI8 2xPhospho [S779(100); S781(100)] | 0.792 | 0.0139 |
| Q8CGB6 | Tensin-2 | Tns2 | Q8CGB6 1xPhospho [S1087(100)] | 0.791 | 0.0027 |
| Q6PE13 | Proline-rich transmembrane protein 3 | Prrt3 | Q6PE13 1xPhospho [S845(100)] | 0.787 | 0.042 |
| Q9QYR6 | Microtubule-associated protein 1A | Map1a | Q9QYR6 1xPhospho [S121(99.9)] | 0.781 | 0.0209 |
| Q8BXR9 | Oxysterol-binding protein-related protein 6 | Osbpl6 | Q8BXR9 1xPhospho [S290(99.9)] | 0.774 | 0.0167 |
| Q61029 | Lamina-associated polypeptide 2, isoforms beta/delta/epsilon/gamma | Tmpo | Q61029 1xPhospho [T159(100)] | 0.756 | 0.0131 |
| P20357 | Microtubule-associated protein 2 | Map2 | P20357 1xPhospho [S1013(98.6)] | 0.751 | 0.0178 |
| Q8CDM8 | Protein FAM160B1 | Fam160b1 | Q8CDM8 1xPhospho [T551(100)] | 0.747 | 0.0004 |
| Q9ERD6 | Ras-specific guanine nucleotide-releasing factor RalGPS2 | Ralgps2 | Q9ERD6 1xPhospho [T290(97.9)] | 0.74 | 0.025 |
| S4R1M9 | Oxysterol-binding protein-related protein 10 | Osbpl10 | S4R1M9 1xPhospho [S32(99.9)] | 0.725 | 0.0394 |
| Q99NE5 | Regulating synaptic membrane exocytosis protein 1 | Rims1 | Q99NE5 1xPhospho [S1448(95.8)] | 0.672 | 0.0348 |
| O35495 | Cyclin-dependent kinase 14 | Cdk14 | O35495 1xPhospho [S95(100)] | 0.671 | 0.0264 |
| P81122 | Insulin receptor substrate 2 | Irs2 | P81122 1xPhospho [S556(100)] | 0.589 | 0.0098 |
| P81122 | Insulin receptor substrate 2 | Irs2 | P81122 1xPhospho [S727(96.8)] | 0.531 | 0.025 |
| P14873 | Microtubule-associated protein 1B | Map1b | P14873 1xPhospho [T340(98.9)] | 0.833 | 0.0326 |
