# Supplementary figures and images for: ZO-1 and IL-1RAP Phosphorylation: Potential Role in Mediated Brain-Gut Axis Dysregulation in Irritable Bowel Syndrome-like Stressed Mice
Source: Int J Med Sci. 2024 Jul 2;21(9):1738–55. doi: 10.7150/ijms.95848 (PMC11241095; doi:10.7150/ijms.95848)

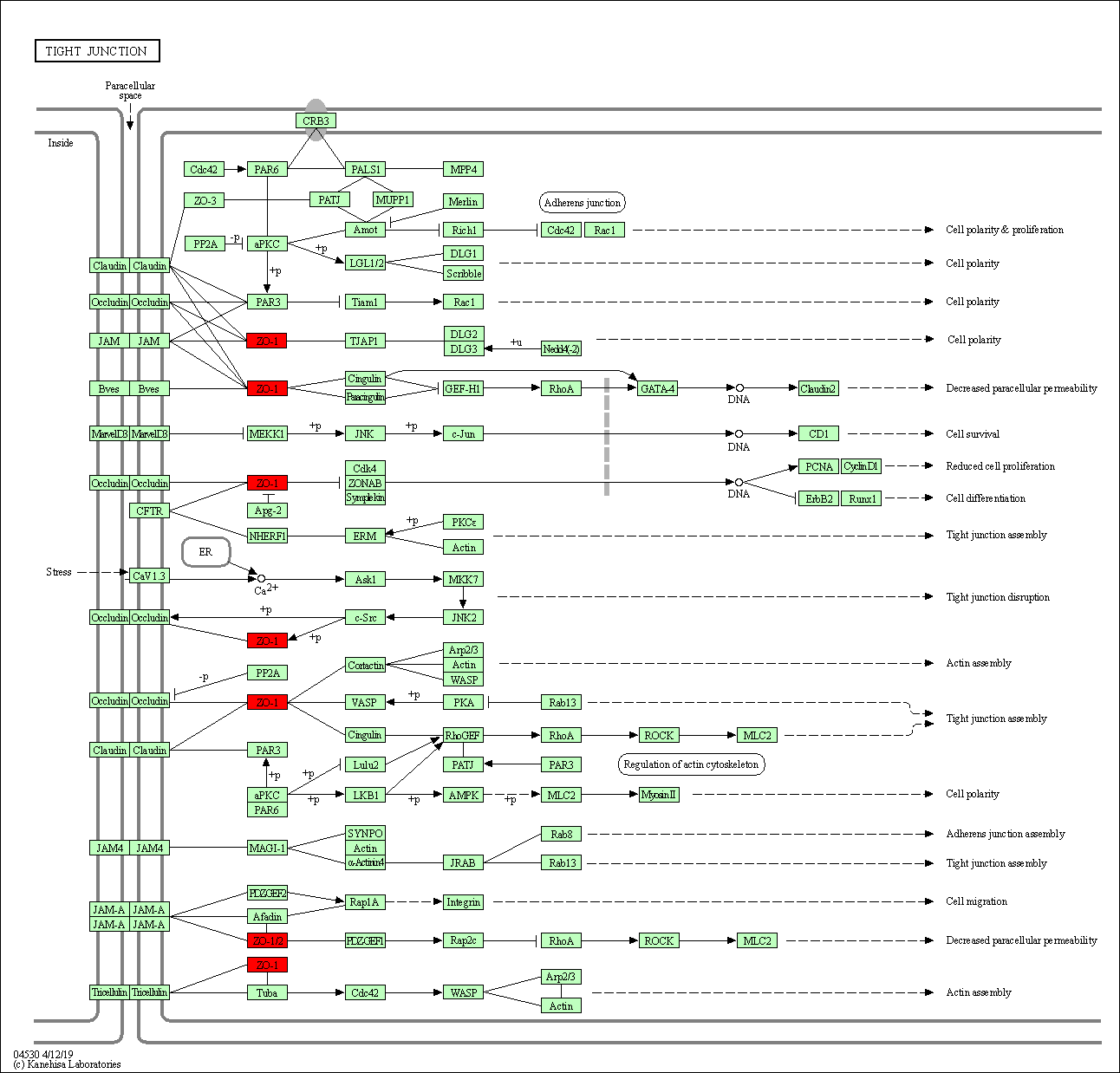

Supplement: Supplementary file 1 — Supplementary figures and tables. [file ijmsv21p1738s1.zip › Supplementary Materials/Figure S1. mmu04530..png]

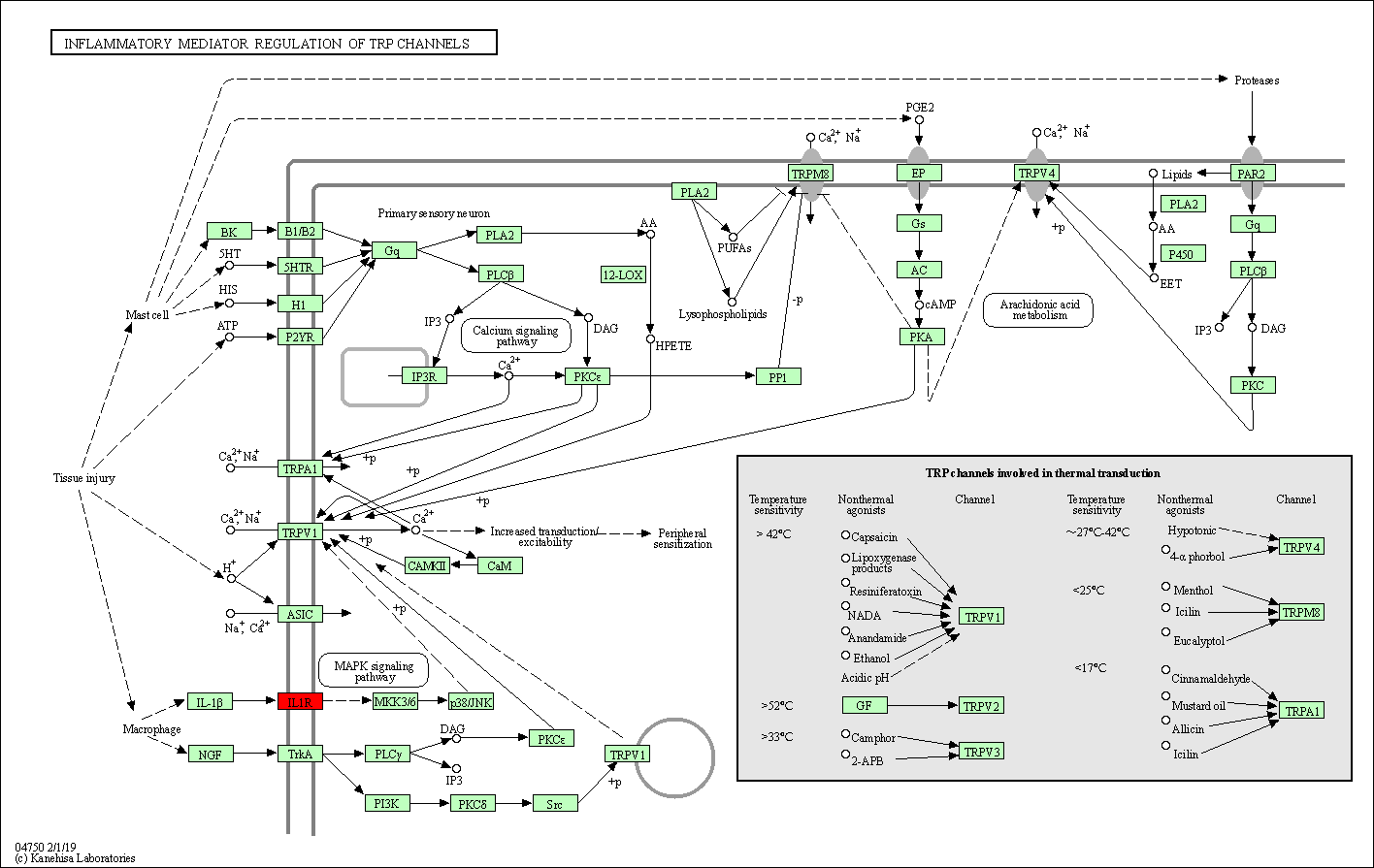

Supplement: Supplementary file 1 — Supplementary figures and tables. [file ijmsv21p1738s1.zip › Supplementary Materials/Figure S2. mmu04750..png]

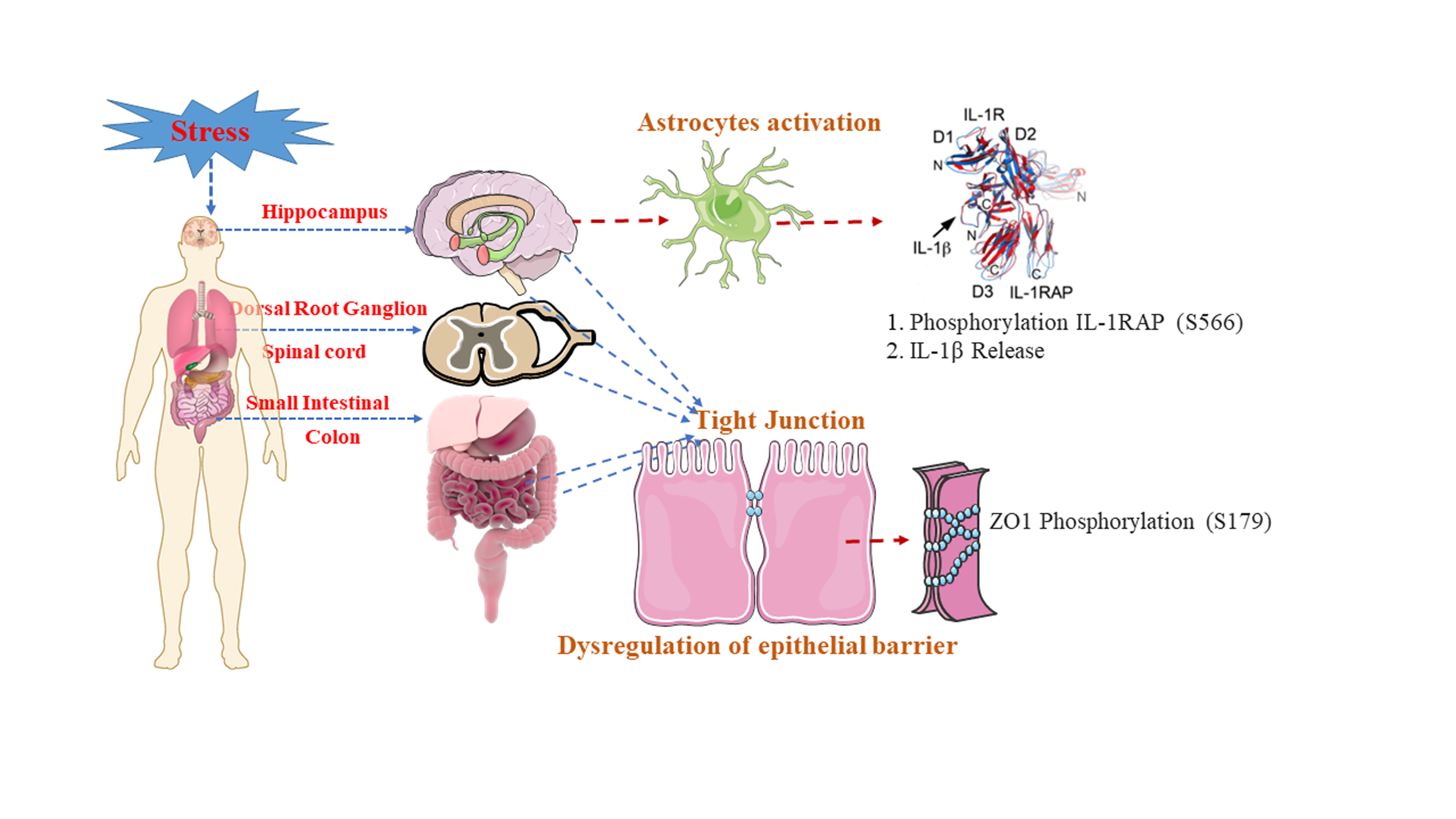

Supplement: Supplementary file 1 — Supplementary figures and tables. [file ijmsv21p1738s1.zip › Supplementary Materials/Graphical Abstract.png]
